# New Deferric Amine Compounds Efficiently Chelate Excess Iron to Treat Iron Overload Disorders and to Prevent Ferroptosis

**DOI:** 10.1002/advs.202202679

**Published:** 2022-08-28

**Authors:** Wenya Feng, Yuanjing Xiao, Chuanfang Zhao, Zhanming Zhang, Wei Liu, Juan Ma, Tomas Ganz, Junliang Zhang, Sijin Liu

**Affiliations:** ^1^ State Key Laboratory of Environmental Chemistry and Ecotoxicology Research Center for Eco‐Environmental Sciences Chinese Academy of Sciences Beijing 100085 P. R. China; ^2^ University of Chinese Academy of Sciences Beijing 100049 P. R. China; ^3^ School of Chemistry and Molecular Engineering East China Normal University 500 Dongchuan Road Shanghai 200241 P. R. China; ^4^ Department of Chemistry Fudan University 2005 Songhu Road Shanghai 200438 P. R. China; ^5^ Department of Medicine David Geffen School of Medicine University of California Los Angeles CA 90095 USA

**Keywords:** deferric amine compound, ferroptosis, iron chelator, iron overload, liver injury

## Abstract

Excess iron accumulation occurs in organs of patients with certain genetic disorders or after repeated transfusions. No physiological mechanism is available to excrete excess iron and iron overload to promote lipid peroxidation to induce ferroptosis, thus iron chelation becomes critical for preventing ion toxicity in these patients. To date, several iron chelators have been approved for iron chelation therapy, such as deferiprone and deferoxamine, but the current iron chelators suffer from significant limitations. In this context, new agents are continuously sought. Here, a library of new deferric amine compounds (**DFAs**) with adjustable skeleton and flexibility is synthesized by adopting the beneficial properties of conventional chelators. After careful evaluations, compound **DFA1** is found to have greater efficacy in binding iron through two molecular oxygens in the phenolic hydroxyl group and the nitrogen atom in the amine with a 2:1 stoichiometry. This compound remarkably ameliorates iron overload in diverse murine models through both oral and intravenous administration, including hemochromatosis, high iron diet‐induced, and iron dextran‐stimulated iron accumulation. Strikingly, this compound is found to suppress iron‐induced ferroptosis by modulating the intracellular signaling that drives lipid peroxidation. This study opens a new approach for the development of iron chelators to treat iron overload.

## Graphical abstract

Here a new deferric amine compound (**DFA1**) is developed, which shows greater efficacy in binding iron than conventional chelators through two molecular oxygens in the phenolic hydroxyl group and the nitrogen atom in the amine with a 2:1 stoichiometry. This compound effectively ameliorates iron overload in diverse murine models and prevents iron‐involved ferroptosis.

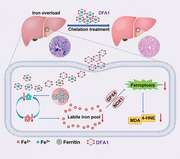

## 1 Introduction

As a key cofactor, iron participates in many fundamental biochemical processes.^[^ ^1^ ^]^ The biological activity of iron largely lies in its ability to act as an electron donor and acceptor while switching between its ferrous and ferric states.^[^ ^2^ ^]^ However, excess iron is detrimental due to oxidative stress giving rise to cellular injury and even leading to iron‐dependent cell death, namely ferroptosis.^[^ ^3^ ^]^ Chronic iron overload occurs in a number of diseases, such as hereditary hemochromatosis (HH), beta‐thalassemia, sickle cell disease (SCD), and myelodysplastic syndromes (MDS). A classical disorder of iron overload, type 1 HH is primarily caused by enhanced dietary iron absorption due to HFE mutations.^[^ ^4^ ^]^ Secondary iron overload results from long term red‐cell transfusion in thalassemia, SCD, MDS, and other disorders but enhanced iron absorption may also contribute.^[^ ^5^ ^]^ Consequently, iron accumulation in diverse organs causes liver injury,^[^ ^6^ ^]^ diabetes mellitus,^[^ ^7^ ^]^ and cardiac dysfunction.^[^ ^8^ ^]^ Therapeutic intervention using iron‐selective chelators represents a critically important strategy to remove excess iron.^[^ ^9^ ^]^

**Figure 1:**
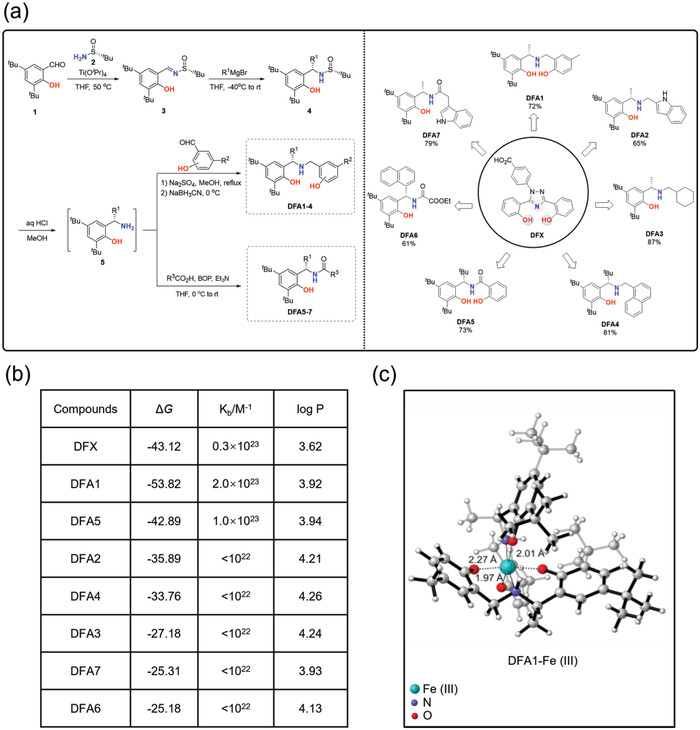
Synthesis and screening of the DFAs library. a) A diagram showing the synthesis of the library of the **DFAs**. A total of 7 **DFAs** were synthesized with formulas in comparison to DFX. b) Values of Δ*G*, K_b_/M^–1^ and log P of newly synthesized compounds with ferric iron. c) The proposed complex structure of **DFA1** in binding with ferric iron.

Iron chelation involves the use of ligands that avidly bind iron and are excreted in stool or urine, thus removing iron from the body to treat iron overload.^[^ ^10^ ^]^ The current iron chelators range from deferoxamine (DFO) to deferiprone (DFP) and deferasirox (DFX) (summarized in Table S1, Supporting Information).^[^ ^11^ ^]^ DFO, which consists of a chain of 3 hydroxamic acids terminating in a free amino acid group, is capable of combining ferric iron at a 1:1 stoichiometry.^[^ ^12^ ^]^ However, the highly hydrophilic structure of DFO results in poor absorption and rapid drug metabolism, so it must be parenterally administered continuously or at least for 8–12 h per day, imposing significant treatment burden for patients and impeding adherence.^[^ ^13^ ^]^ There have been several attempts to prolong the half‐lives to improve iron elimination efficiency and to reduce the toxicity by exploiting macromolecules or nanoparticle (NP), such as dendrimers, polymer conjugation, and amphiphilic copolymer NPs. However, their biodistribution patterns and elimination pathways are not fully defined yet.^[^ ^11^, ^14^ ^]^ Although DFP is less burdensome to patients,^[^ ^15^ ^]^ its adverse effects restrict its widespread applications.^[^ ^16^ ^]^ Recently, to minimize the influence of metabolism, a sacrificial site for glucuronidation was introduced in DFP for greater iron scavenging efficacy.^[^ ^9b^ ^]^ Moreover, DFX utilizes a triazolyl nitrogen and two phenolic oxygens as donor groups (highlighted in red and blue, **Figure** **1**a, in the right panel), which coordinate with iron to form a 2:1 complex. Although the recently approved chelator DFX has improved compliance compared to DFO and DFP, the oral administration of DFX also manifests significant adverse effects, such as gastrointestinal and renal toxicities.^[^ ^17^ ^]^ Nonetheless, tremendous efforts are seen in the literature in developing different chemicals to coordinate with iron.^[^ ^18^ ^]^ With the aim of developing more effective methods for delivering iron chelators, new chelators with greater therapeutic efficacy and less toxicity are very much needed.

Taking into account the structural attributes and practical limitations of the current iron chelators, we here designed a library of new deferric amine compounds (**DFAs**) with adjustable skeleton and flexibility, where the substituents at the phenol moiety would prevent its oxidization to quinone (Figure 1a). We here hypothesized that the oxygen and nitrogen combination in **DFAs** confer the binding with iron in highly stable O, N, O‐Fe complexes. After screening, compound **DFA1** out of the library showed a remarkable efficacy in chelating excess iron in vitro and in vivo, even greater than conventional chelators. Moreover, this compound prevented ferroptosis induced by iron overload. Together our data indicate that this new **DFA** represents a promising lead for further development.

## 2 Results

### 2.1 Molecular Design, Synthesis, and Characterization of DFAs

In search of new iron chelators, we designed and synthesized a library of 7 **DFAs** by incorporating differential groups on the parental compounds with adjustable skeleton and flexibility. These **DFAs** were prepared with a purity greater than 95%, as determined by high performance liquid chromatography (HPLC). The synthetic route of the **DFAs** is summarized in Figure 1a, starting from 3,5‐di‐tert‐butyl‐2‐hydroxybenzaldehyde and chiral sulfinamide. The structural formula is shown in Figure 1a (in the right panel), where the compounds are proposed to coordinate with ferric ion through the oxygen atom in the phenolic hydroxyl group and the nitrogen atom of amine (highlighted in red and blue).

Afterward, density functional theory (DFT) calculations were carried out to predict the iron binding capability. As shown in Figure 1b, the binding energies (Δ*G*, minus) of tridentate ligands (**DFA1**, **DFA5,** and DFX) were lower than those of bidentate ligands (**DFA2**, **DFA3**, **DFA4**, **DFA6,** and **DFA7**). By contrast, replacing amine with amide weakened their ferric binding (Δ*G* of **DFA5** was higher than **DFA1,** and **DFA7** was higher than **DFA2**). Among them, the Δ*G* values of **DFA1** were lower than that of DFX (−53.82 versus −43.12 kcal mol^−1^) (Figure 1b). Furthermore, the binding constants (K_b(iron‐ligands)_) of **DFAs** with Fe(III) were studied with the fluorescence displacement method (Figure 1b). Among 7 **DFAs**, only **DFA1** and **DFA5** were found to be able to effectively compete with calcein to bind Fe(III). Compared with DFX, the K_b(Fe(III)‐DFA1)_ was 2.0 × 10^23^ m ^–1^ and the K_b(Fe(III)‐DFA5)_ was 1.0 × 10^23^ m ^–1^, respectively, indicative of stronger ferric binding than DFX (K_b(Fe(III)‐DFX)_ was 0.3 × 10^23^ m ^–1^). The ferric binding mode for **DFA1** is shown in Figure 1c. Meanwhile, the octanol‐water partition coefficient (log P) values of **DFA1** and **DFA5** were higher than DFX^[^ ^19^ ^]^ (Figure 1b), implying that the introduction of tert‐butyl strongly increased the lipophilicity. The higher lipophilicity of **DFAs** could facilitate their transport across the plasma membrane, where they could exhibit the iron‐chelating ability inside the cell.^[^ ^20^ ^]^

### 2.2 Screening of Synthesized Compounds for the Binding of Ferric Iron

**Figure 2:**
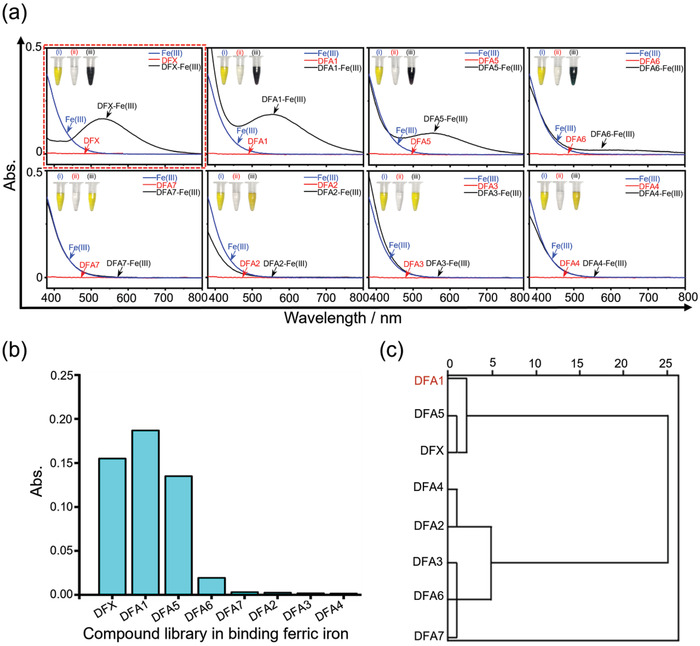
Screening of the DFAs in binding ferric iron. a) The UV–vis absorption spectra of **DFAs** upon incubation with FeCl_3_. b) Quantification of UV–vis absorption peaks at 550 nm for DFX and 7 **DFAs** after binding to ferric iron. c) Dendrogram display from an unsupervised hierarchical cluster with Ward's method based on the values of Δ*G*, K_b_/M^–1^, log P, and UV–vis absorption.

To assay the iron chelating capability of our synthesized compounds, we probed their binding to ferric iron using UV–visible absorption spectroscopy. As shown in **Figure** **2**a, **DFA1** and **DFA5** immediately yielded black‐purple substances upon the addition of ferric iron, generating a new ultraviolet absorption peak at 550 nm. Meanwhile, **DFA6** also quickly reacted with ferric iron, giving rise to dark‐green products and a new absorption peak at 550 nm (Figure 2a). However, **DFA2**, **DFA3, DFA4,** and **DFA7** did not react with ferric iron, as no color change was observed in response to ferric iron (Figure 2a,b). Furthermore, the metal ion selectivity of **DFAs** was screened. As shown in Figure S1, Supporting Information, the absorption reactions of **DFAs** were selective for Fe(III) over other abundant cellular alkaline earth metal ions, such as Mg(II), and other biologically relevant transition metal ions, such as Cu(II), Zn(II), Co(II), Ni(II) and Mn(II). Differently, **DFA1** and **DFA5** manifested minimal absorption toward Fe(II). Collectively, these results indicated that our compounds possessed high selectivity for Fe(III) over the other metals tested here. Hierarchical cluster analyses were performed to quantify the overall correlation between the iron chelating efficacy and their chemical properties (including the UV absorption, K_b(iron‐ligands)_, Δ*G*, and log P) through Ward's agglomeration using log‐transformed normalized values of various factors, as described.^[^ ^21^ ^]^ As depicted in Figure 2c, **DFA1** clustered separately from the others, in support of the above experimental data. To this end, **DFA1** may therefore be a desirable lead for iron chelation and selected **DFA1** for further detailed evaluation.

### 2.3 Assessment of Iron Chelating Efficacy In Vitro Using Hepatocyte Cell Lines

**Figure 3:**
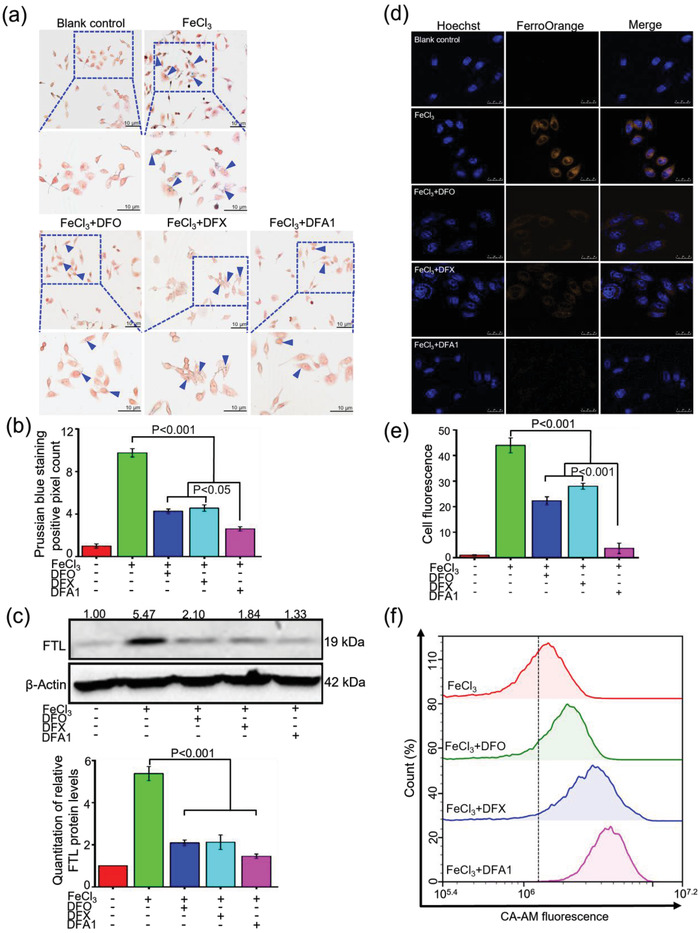
Survey of iron chelation efficacy for DFAs in HepG2 cells. a) Prussian blue staining images of HepG2 cells after 3 h per incubation with FeCl_3_ at 100 µm, followed by treatment with DFO, DFX, and **DFA1** at 20 µm for 12 h. The lower panels show the enlarged images. The quantified data of positive pixel counts of Prussian blue staining in HepG2 cells are shown in (b) (*n* = 3). c) Western blotting determination of FTL protein content in the above‐treated cells. The ratios of FTL to *β*‐actin were calculated, and the ratio of in the blank control is defined as 1.00. The corresponding ratios are presented above the autoradiograms. Quantified data for multiple biological replicates are shown in the lower panel (*n* = 3). d) Representative confocal microscopy images showing intracellular ferrous iron in the above‐treated cells, as reflected by FerroOrange probes. Cells were stained with FerroOrange probes (in brown color) to visualize the intracellular ferrous iron. Hoechst 33342 was used to stain nuclei (in blue color). Scale bar, 25 µm. Quantified data of cellular fluorescence were shown in (e) (*n* = 3). f) Determination of the intracellular ferrous iron concentration, namely LIP, with the Ca‐AM probes in the above‐treated HepG2 cells through flow cytometry.

Next, the cytotoxicity of this compound was determined, as shown in Figure S2, Supporting Information. The cell counting kit‐8 (CCK‐8) assay showed no observable toxicity to human hepatoma HepG2 cells, mouse hepatocyte NCTC cells, mouse myoblast progenitor cell line (C2C12), mouse fibroblast L929 cells, and human embryonic kidney cell line HEK‐293T cells below 50 µm (Figure S2a–e, Supporting Information), and propidium iodide (PI) staining of HepG2 and NCTC further corroborated this finding (Figure S2f,g, Supporting Information). To interrogate the iron removal efficacy, cells were first replenished with 100 µm FeCl_3_ for 3 h to induce cellular iron accumulation prior to compound treatment. Intracellular iron content was reflected by Prussian blue staining, as shown in **Figure** **3**a,b, **DFA1** greatly reduced intracellular iron accumulation in FeCl_3_‐pretreated cells by 73% relative to untreated control (*P* < 0.001). Furthermore, L‐ferritin light (FTL) level after **DFA1** treatment remarkably diminished in FeCl_3_‐pretreated HepG2 cells in comparison to untreated cells. (Figure 3c). In addition to reducing ferric iron, non‐fluorescent FerroOrange probes were used to detect intracellular ferrous irons. As shown in Figure 3d,e, in contrast to the massive yellow punctate distribution in FeCl_3_‐pretreated cells, the fluorescence intensity declined by 73% for **DFA1**, 48% for DFO and 46% for DFX, respectively (*P* < 0.001). Intracellular ferrous iron was also determined by flow cytometry analysis using Calcein‐AM (Ca‐AM). As shown in Figure 3f, **DFA1** increased the fluorescent intensity by 117% in FeCl_3_‐pretreated cells, and DFO and DFX increased the fluorescent intensity by 47% and 79%, respectively, compared to untreated cells. Additionally, similar results were obtained in NCTC cells, as shown in Figure S3, Supporting Information. Collectively, these results suggested that **DFA1** could efficiently chelate intracellular iron in vitro and revealed greater efficacy than DFO and DFX.

### 2.4 Parenteral Administration of Compound DFA1 Alleviated Iron Overload in Animal Models

In vivo efficacy was assessed in different mouse models of iron loading. *Hfe^–/–^* mice were treated with **DFA1** administered by i.v. injection (Figure S4a, Supporting Information), greatly reduced the hepatic and splenic iron content relative to untreated controls, as characterized by the tissue iron staining (Figure S4b, Supporting Information). Moreover, this reduction was further confirmed by the FTL levels in the livers of treated mice compared to untreated mice (Figure S4c,d, Supporting Information, *P* < 0.05). DFO was used as a control, but it was less effective in reducing the iron burden in *Hfe^–/–^* mice than **DFA1** (Figure S4, Supporting Information).

**Figure 4:**
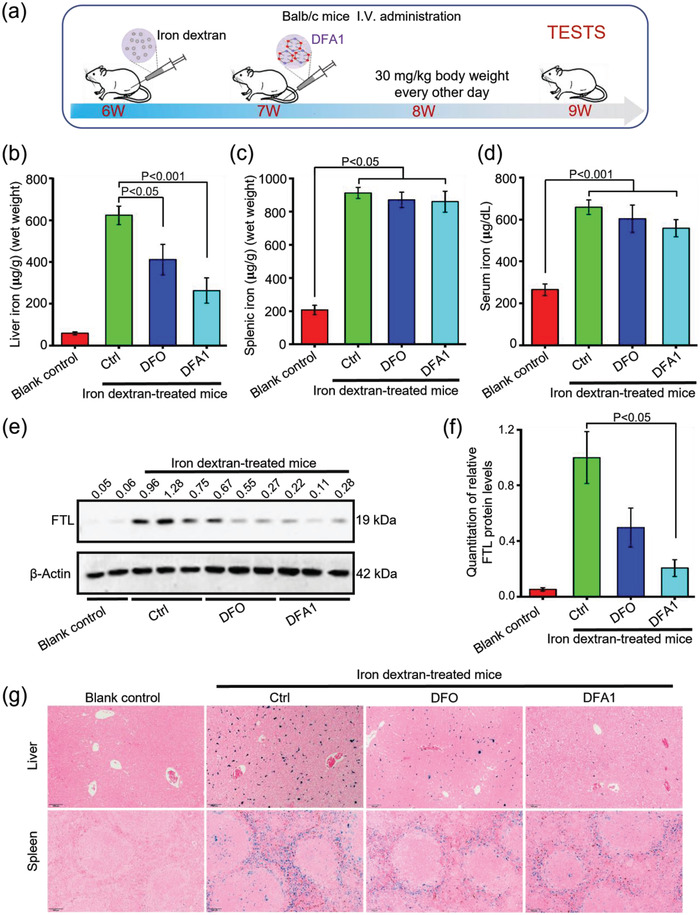
Parenteral DFA1 relieved iron overload induced by iron dextran in wild‐type mice. a) A diagram of the experimental design. Here, wild‐type mice of 6‐weeks old were subjected to intraperitoneal injection of iron dextran at a dose of 150 mg kg^−1^ body weight for 1 week, followed by administration with DFO and **DFA1** at a dose of 30 mg kg^−1^ body weight every other day for 2 weeks. b) Hepatic, c) splenic, and d) serum iron was then assayed (*n* = 5–6). Meanwhile, e) FTL protein levels were determined by Western blot analysis in liver specimens, and quantified data of FTL proteins relative to the internal control are shown in (f) (*n* = 3). g) Tissue iron staining of liver and spleen sections with Prussian blue (in blue). Scale bar, 100 µm.

Furthermore, another iron overload mouse model was established through iron dextran administration, as previously reported.^[^ ^22^ ^]^ As shown in **Figure** **4**, significant iron accumulation was demonstrated in these mice after iron dextran administration, including in the liver, spleen, and serum. As illustrated in Figure 4a, **DFA1** was given to these mice every other day for 2 weeks. Consistently, **DFA1** reduced the iron load in this mouse model more efficiently than DFO, as evidenced by tissue iron measurements, FTL concentrations, and iron staining (Figure 4b–g).

As previously documented,^[^ ^23^ ^]^ DFO suffers from a short half‐life ≈5–15 min, and therefore requires long periods of subcutaneous infusion over 8–12 h per day, 5–7 days a week, to achieve adequate concentrations of the drug, and its use is burdensome for patients (Table S1, Supporting Information). We, therefore, determined the half‐life of **DFA1** in comparison to DFO (Figure S5, Supporting Information). DFO had a short half‐life (t_1/2_) ≈0.14 ± 0.74 h in sera, whereas the half‐life for **DFA1** was much longer at 2.01 ± 0.08 h (Figure S5b,c, Supporting Information, *P* < 0.05). Moreover, the area under the curve (AUC) for **DFA1** was calculated to be 299.00 ± 38.24 mg h L^−1^ in contrast to 6.289 ± 1.85 mg h L^−1^ for DFO (Figure S5c, Supporting Information, *P* < 0.001). The higher AUC value indicates the greater drug exposure after a single dose.^[^ ^24^ ^]^

### 2.5 Oral Administration of DFA1 Mitigated Iron Overload in HFE Mice

**Figure 5:**
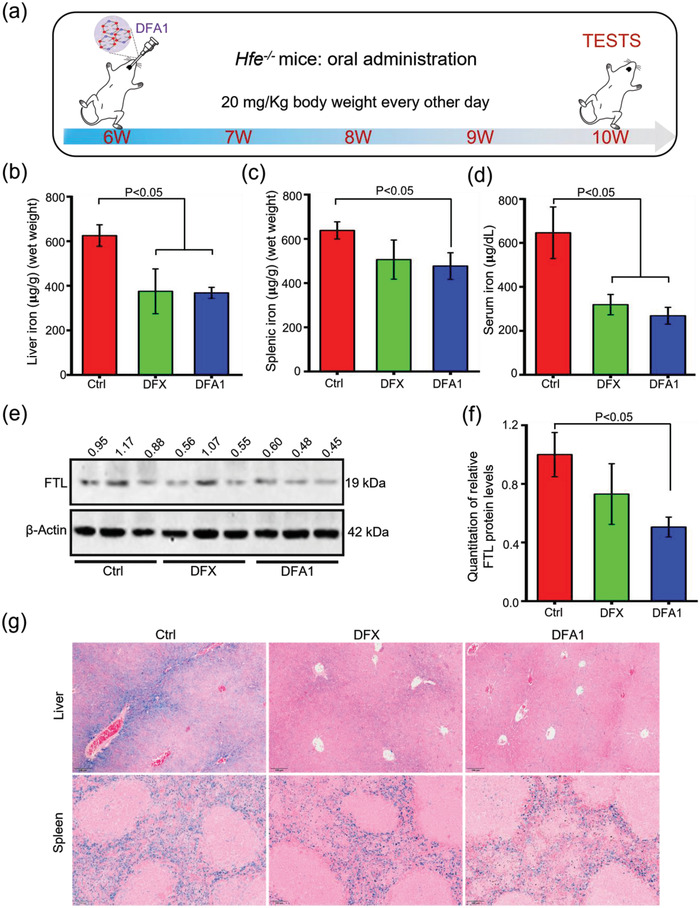
Oral administration of DFA1 alleviated iron overload in *Hfe^–/–^* mice. a) A diagram depicting the experimental design. After oral treatment for *Hfe^–/–^* mice with DFX and **DFA1** at a dose of 20 mg kg^−1^ body weight every other day for 4 weeks, b) hepatic, c) splenic and d) serum iron mass was then determined (*n* = 5–6). e) Hepatic FTL levels were assessed by Western blot analysis in liver specimens, and quantified data relative to the internal control are shown in (f) (*n* = 3). g) Tissue iron staining of liver and spleen sections with Prussian blue (in blue color). Scale bar, 100 µm.

The chronic regimen of i.v. administration is problematic for most patients, often resulting in poor patient compliance. Thus, oral drugs present an attractive option for most types of iron overload disorders.^[^ ^25^ ^]^ The iron chelating efficacy after oral administration of our compound was examined in *Hfe^–/–^* mice (**Figure** **5**a). Like after i.v. administration (Figure S4, Supporting Information), the liver iron concentration was significantly reduced by 40% upon oral administration of **DFA1** relative to untreated mice (Figure 5b, *P* < 0.001), and splenic iron content was also reduced (Figure 5c, *P* < 0.05). Moreover, serum iron concentration diminished by nearly 60% in *Hfe^–/–^* mice relative to untreated mice (Figure 5d, *P* < 0.05). In agreement with the direct hepatic iron measurement, the FTL levels in the livers of **DFA1‐**treated mice were decreased by 44%, compared to untreated mice (Figure 5e,f, *P* < 0.05). Iron staining of liver and spleen sections confirmed the above results (Figure 5g). Different from the changes in liver and spleen iron concentrations, there was no significant change in iron content in the heart, lung, and kidney. This may be due to the fact that the disruption of *Hfe* gene predominantly resulted in severe liver iron deposition; however, iron mass in the heart, lung, and kidney is not significantly changed, as demonstrated previously.^[^ ^26^ ^]^

### 2.6 DFA1 Greatly Prevented Iron‐Induced Ferroptosis In Vitro and In Vivo

**Figure 6:**
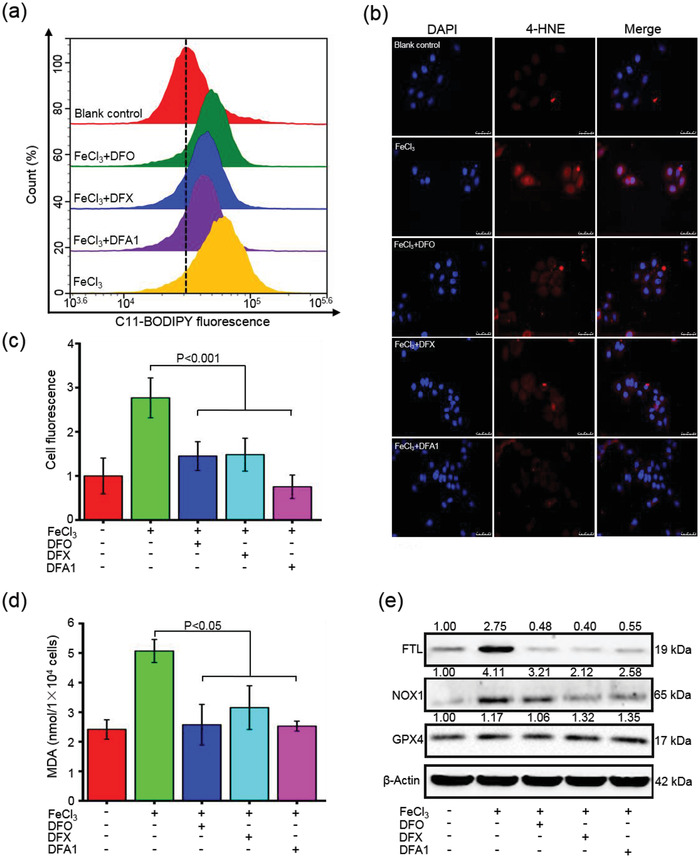
DFA1 alleviated iron‐induced ferroptosis in vitro. a) Determination of lipid peroxidation levels in HepG2 cells with pretreatment of FeCl_3_ at 100 µm for 12 h, followed by treatment with DFO, DFX, and **DFA1** at 20 µm for 12 h. Thereafter, lipid peroxidation was assessed by C11‐BODIPY^581/591^ probes through flow cytometry. b) Representative images of 4‐HNE immunofluorescent staining (in red color) through fluorescent microscopy. DAPI was used to stain nuclei (in blue). Quantification of cellular 4‐HNE fluorescence was shown in (c) (*n* = 3). d) Cellular MDA content was assayed in the above‐treated HepG2 cells (*n* = 4), and e) the protein levels of FTL, NOX1, and GPX4 were analyzed by Western blotting. The ratios of target proteins to the internal control are shown above the autoradiograms.

Since redox‐active ferrous iron catalyzes the generation of lipid peroxidation through Fenton reaction, lipid peroxidation represents an indication of the initiation of ferroptosis.^[^ ^27^ ^]^ We therefore examined anti‐ferroptosis effects of **DFA1** in vitro and in vivo. Here, lipid peroxidation in live cells was detected using the C11‐BODIPY^581/591^ reagent.^[^ ^28^ ^]^ As shown in **Figure** **6**a, FeCl_3_ treatment triggered substantial lipid peroxidation in HepG2 cells, as characterized by an increase of fluorescent intensity of C11‐BODIPY^581/591^ relative to untreated cells. However, the increase was largely reversed by DFO, DFX, and **DFA1** by 20%, 27%, and 29%, respectively (Figure 6a). The content of 4‐hydroxynonenal (4‐HNE), the end product of lipid peroxidation,^[^ ^29^ ^]^ was evaluated by immunofluorescent microscopy. There was minimal background immunofluorescence for 4‐HNE in blank control cells, but FeCl_3_ pretreatment greatly enhanced 4‐HNE staining. Strikingly, all these iron chelators markedly inhibited the 4‐HNE staining relative to untreated cells, and **DFA1** diminished 4‐HNE content to a greater extent than DFO and DFX (Figure 6b,c, *P* < 0.05). In addition, the levels of MDA, another product of lipid peroxidation,^[^ ^30^ ^]^ were greatly reduced in FeCl_3_‐pretreated cells following incubation with each of the chelators (Figure 6d, *P* < 0.05).

To validate the above findings on the anti‐ferroptosis effects of **DFA1**, we looked at the protein levels of key regulators of ferroptosis.^[^ ^31^ ^]^ Consistent with the above findings, FeCl_3_ elevated the intracellular FTL in HepG2 cells, and conversely, these chelators significantly reduced the FTL levels (Figure 6e). Meanwhile, FeCl_3_ stimulated the concentration of NADPH oxidase 1 (NOX1) by more than twofold relative to the blank control cells, but **DFA1** reversed the induction of NOX1 by FeCl_3_ (Figure 6e). **DFA1** elevated the levels of glutathione peroxidase 4 (GPX4) in FeCl_3_‐pretreated cells (Figure 6e), indicative of enhanced protection from ferroptosis. Notably, **DFA1** induced greater changes in NOX1 and GPX4 in HepG2 cells than did DFO and DFX (Figure 6e). Moreover, similar anti‐ferroptosis effects were demonstrated in NCTC cells treated with **DFA1** (Figure S6, Supporting Information).

**Figure 7:**
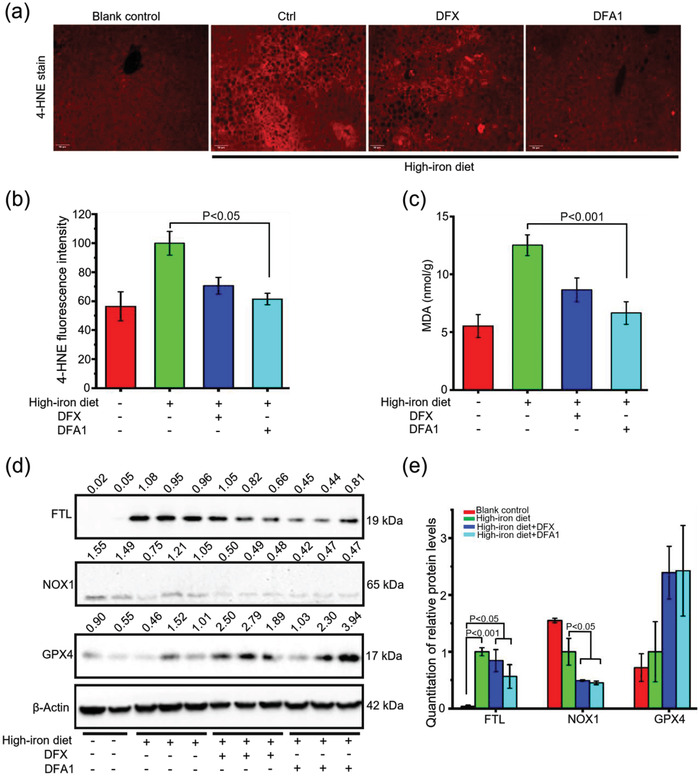
DFA1 mitigated liver cell ferroptosis in mice fed high‐iron diet. a) Representative immunofluorescent images of liver sections with staining of 4‐HNE from mice under high‐iron diet with or without oral administration of DFX and **DFA1** at a dose of 20 mg kg^−1^ body weight every other day for 4 weeks. Scale bar, 100 µm. b) Quantification of hepatic 4‐HNE fluorescence by calculating 4 fields from 2 biological replicates. c) Hepatic MDA levels were measured (*n* = 6–8). d) Western bolt analysis of hepatic FTL, NOX1, and GPX4 levels in the above‐treated mice, and quantified data relative to the internal control are shown in (e) (*n* = 3).

Furthermore, the anti‐ferroptosis effect was examined in mouse models with hepatocyte ferroptosis induced by high‐iron diet. In agreement with previous reports,^[^ ^32^ ^]^ high‐iron diet induced hepatic ferroptosis, as characterized by the increase of 4‐HNE staining and MDA measurement in the liver relative to the blank control. Intriguingly, **DFA1** was inhibited by more than 50% the increase of 4‐HNE and MDA in mice fed a high‐iron diet compared to untreated mice (**Figure** **7**a–c). In support of these findings, **DFA1** treatments also greatly reduced the NOX1 level and conversely elevated the GPX4 level in the livers of mice on high‐iron diet, in parallel to the changes in FTL levels (Figure 7d,e).

### 2.7 DFA1 Showed Great Biosafety in Animals

As reported previously, DFX could induce significant nephrotoxicity.^[^ ^11^, ^17^ ^]^ To examine whether the administration of **DFA1** was associated with any kidney toxicity, we compared **DFA1** with DFX in a mouse model under a high‐iron diet that induces renal toxicity. DFX, but not **DFA1**, caused observable renal toxicity in these mice, as reflected by the elevation of creatinine (Cr) and beta‐2 microglobulin (*β*2M) (*P* < 0.05) (Figure S7, Supporting Information). By contrast, **DFA1** did not increase the Cr and *β*2M levels, indicating greater bio‐compatibility. Additionally, **DFA1** overall showed good bio‐compatibility in our tests, as evidenced by the lack of significant changes in various serum markers and histological examination in diverse mouse models (Figures S8–S10, Supporting Information). Furthermore, no significant toxicity was found at higher doses (e.g., 50 or 100 mg kg^−1^ body weight) in wild‐type mice, as evidenced by histological examination (Figure S11a, Supporting Information). Compared with the control group, DFX slightly increased the levels ALT and AST levels at 100 mg kg^−1^ body weight, but not at 50 mg kg^−1^ body weight (Figure S11b,c, Supporting Information). Differently, our compound **DFA1** incurred little change in ALT and AST levels, showing greater biosafety. Collectively, these results indicated that **DFA1** has considerable promise as an efficient oral iron chelator.

## 3 Discussion

We here reported that 7 **DFAs** were synthesized. These compounds have a flexible structure with adjustable skeleton, which can improve iron chelating efficacy and hydrophobicity by adding different groups. Oxygen and nitrogen atoms, as electron pair donor, donate electron pair to ferric iron, and form stable complex with a stoichiometric coefficient 2:1. Strikingly, the newly synthesized compounds prevent their further oxidation due to the tert‐butyl group at the ortho and para position of the phenolic hydroxyl group. After DFT calculations, we found that the binding energy of tridentate ligands was lower than those of bidentate ligands, meaning that the tridentate ligands donate 3 electron pairs for ferric and show stronger ferric binding than bidentate ligands.

We further examined the impacts of different substituent groups on coordination. As shown in Figure 1a, when the amine group was replaced by the amide group (Δ*G* of **DFA5** was higher than **DFA1**, and Δ*G* of **DFA7** was higher than **DFA2**), this change reduced the electron density from the amide nitrogen due to the electron‐withdrawing effect of the carbonyl group,^[^ ^33^ ^]^ leading to diminished chelation efficacy of such compounds with iron. Furthermore, when one phenol moiety was replaced by naphthyl (compound **DFA4**), the coordination could not take place. In fact, the coordination with iron required a certain spatial distance and a small steric hindrance, and some modifications would alter the optimal spacing necessary for binding, resulting in reduction of the iron binding capability.^[^ ^33^, ^34^ ^]^ These observations collectively suggested that the two oxygen atoms of the phenolic hydroxyl group and the nitrogen atom on the amine were necessary to coordinate iron.

Since hepatocytes are the major cell type for iron storage,^[^ ^35^ ^]^ HepG2 and NCTC cells were employed here. Cytotoxicity and PI staining indicate great cyto‐compatibility in vitro. Prussian blue reacts with ferric iron to produce blue precipitate, which reflected intracellular iron content. **DFA1** treatment greatly reduced intracellular iron blue precipitate in FeCl_3_‐pretreated HepG2 and NCTC cells. Furthermore, ferritin level was measured by western blotting as a reporter of intracellular iron storage. Analogous to the iron staining results, **DFA1** remarkably diminished the intracellular FTL protein levels in FeCl_3_‐pretreated HepG2 and NCTC cells in comparison to untreated cells. Intracellular ferrous iron was also determined by non‐fluorescent FerroOrange probes, as these probes can specifically react with ferrous iron to emit intensive fluorescence at 572 nm.^[^ ^31^ ^]^ The fluorescence intensity after **DFA1** treatment was reduced. To substantiate the above observations, intracellular ferrous iron was also determined by flow cytometry analysis using Ca‐AM, whose fluorescence is quenched by ferrous iron,^[^ ^36^ ^]^ **DFA1** increased the fluorescent intensity than DFO and DFX. These data indicated that **DFA1** could greatly reduce the intracellular ferrous iron bounden to a greater extent than DFO and DFX.

The capacity of **DFA1** to remove iron in vivo was assessed in several animal models of iron loading. Disruption of HFE genes (*Hfe^–/–^*) causes HH, resulting in severe liver iron deposition.^[^ ^37^ ^]^ Treatment of *Hfe^–/–^* mice i.v. with **DFA1**, greatly reduced hepatic and splenic iron content relative to untreated controls. Analogous to the above results, administration of iron dextran, these animals accumulated significant amounts of iron in the liver, spleen, and serum. Treatment with **DFA1** reduced the iron load more efficiently than DFO, as shown by tissue iron measurements, FTL concentrations, and iron staining. Notably, iron accumulated in macrophages in the liver of iron dextran‐treated mice, in stark contrast to exclusively iron accumulation in hepatocytes in the *Hfe^–/–^* mice. Nevertheless, our findings indicated that **DFA1** could efficiently mitigate iron load in both hepatocytes and macrophages in the liver. In this study, **DFA1** provides favorable significate chelation of extracellular iron and decreases toxicities significantly. Of note, our compound showed an overall greater efficacy in reducing iron accumulation in *Hfe^–/–^* mice than that of DFX after oral administration.

Given that iron plays an indispensable role in driving intracellular lipid peroxidation and execution of the ferroptosis program, iron chelation has been proposed as a means to suppress iron‐dependent ferroptosis by reducing the free labile iron in cells.^[^ ^3^, ^38^ ^]^ Iron‐dependent ferroptosis has been demonstrated in iron overload cells and high‐iron diet mice as evidenced by a reduced glutathione level and increased lipid peroxidation. Strikingly, **DFA1** inhibits lipid peroxidation and protects cells from oxidative stress‐associated damage following iron chelation. Overall, **DFA1** manifested remarkable anti‐ferroptosis effects, at least partially dependent on iron chelation.

In conclusion, we designed novel **DFAs** with adjustable skeleton and flexibility in coordinating iron. Our work revealed that two molecular oxygen atoms in the phenolic hydroxyl group and the nitrogen atom in the amine played a key role in chelating iron, and compound **DFA1** of the library showed greater iron binding than other compounds or the conventional therapeutic chelators. **DFA1** effectively removed excess iron in vitro and in mouse models of iron overload, manifesting remarkable efficacy in ameliorating iron‐associated damage including ferroptosis. Together, our compound **DFA1** improves therapeutic efficacy and mitigates chelator‐associated adverse effects after i.v. and oral administration. **DFA1** is an attractive lead compound for further therapeutic development.

## 4 Experimental Section

### Reagents

DFO, DFX, ferrous chloride (FeCl_2_), ferric chloride (FeCl_3_), and iron dextran were obtained from Sigma Aldrich (USA). Ca‐AM was purchased from the AAT bioquest (USA). CCK‐8, Cremopho EL, and MDA assay kits were purchased from Solarbio (Beijing, China). Serum iron assay kit, alanine aminotransferase assay kit (ALT), aspartate aminotransferase assay kit (AST), lactate dehydrogenase assay kit (LDH), and Cr assay kit were obtained from the Nanjing Jiancheng Bioengineering Institute (Nanjing, China). C11‐BODIPY^581/591^ probes were obtained from Invitrogen (USA). FerroOrange probes were purchased from Dojindo Molecular Technologies, Inc. (Japan). Methanol and acetonitrile were purchased from Sinopharm Chemical Reagent Co., Ltd (Shanghai, China). Dulbecco's modified Eagle's medium (DMEM), RPMI 1640, and phosphate buffered saline (PBS) were purchased from Corning (USA). Fetal bovine serum (FBS) was purchased from Gibco Inc. (USA).

### Procedures for the Synthesis of DFAs

Detailed description of the general procedures for the synthesis of **DFAs** is provided in the Supporting Information.

### Spectrometry of Analysis of Iron Binding for Synthesized Compounds

To determine whether **DFAs** bind with iron and other metals, synthesized compounds were dissolved in MeOH solution at a concentration of 4.0 mg mL^−1^, followed by mixing with FeCl_3_, FeCl_2_, CuCl_2_, ZnCl_2_, CoCl_2_, NiCl_2_·6H_2_O, MgCl_2_·6H_2_O and MnCl_2_·4H_2_O at a concentration of 1.0 mg mL^−1^, individually. Then, the absorbance value was measured after 50 times dilution through a UV–vis spectrophotometer DU‐800 (Backman, USA).

### Measurement of the Fe(III)‐Binging Affinity with DFAs

The binding affinity of Fe(III) with ligands (K_b(Fe(III)‐ligands)_) was measured with the fluorescence displacement method using a calcein fluorescent agent. Calcein can be used as a fluorescence probe because the fluorescence quenches upon interaction with iron. When ligands competed to bind iron from calcein, the fluorescence quenching of calcein‐iron was inhibited. In the solution of PBS (100 mm, pH 7.4), the concentration of calcein and Fe(III) were maintained at 1 and 20 µm, respectively. IC50 was defined as the 50% inhibitory concentration of each ligand. The binding constants between each ligand and Fe (III) were calculated with the following equation:^[^ ^20^, ^39^ ^]^

(1) $$K_{d}=\frac{IC_{50}}{\left[1+\left(\mathrm{probe}\right)/K_{\mathrm{probe}\left(\mathrm{calcein}\right)}\right]}\;$$

(2) $$K_{b\left(\mathrm{Fe}\left(\mathrm{III}\right)-\mathrm{ligand}\right)}=\frac{1}{K_{d}}\;$$

where the (probe) is the concentration of calcein at 1 µm, and the intrinsic calcein‐binding constant of Fe(III), *K*_b(Fe(III)‐calcein)_ is 10^24^ m ^–1^. *K*_probe(calcein)_ is the dissociation constant for the intercalation of Fe(III) with calcein, and *K*_probe (calcein)_ is 1/10^24^ m.

### Determination of Octanol‐Water Partition Coefficients

Log P was measured based on the 1‐octanol/water system, as described.^[^ ^40^ ^]^ Briefly, equal proportions of octanol and water were balanced for 12 h. Then, DFX and 7 **DFAs** were dispersed in balanced octanol and water, followed by vigorous vortex for 3 min, and then allowed to stand for 15 min, respectively. Afterward, 1.0 mL of the octanol or aqueous phase was transferred to a quartz cuvette and the optical density (OD) of the solution was measured on a UV–vis spectrophotometer (DU‐800) (Backman, USA).

### Computational Methods

To predict the ferric binding ability of newly designed ligands, the DFT computation analysis was carried out. All the structures of newly synthesized ligands, DFX, and their ferric complexes were optimized with B3LYP methods, a mixed basis set of LANL2DZ for Fe (III) and 6‐31G (d,p) for other atoms, as described.^[^ ^41^ ^]^ To compute thermodynamic parameters, the frequency analyses were also performed at the same theoretical level. The solvent effects were considered by computing single point energy on the optimized structures with M06 method,^[^ ^42^ ^]^ as follows, 6‐311++G (d,p) basis set for C, H, O, and N atoms, SDD basis set for Fe (III),^[^ ^43^ ^]^ and SMD solvation model.^[^ ^44^ ^]^ All the computation was carried out with Gaussian 09 software.^[^ ^45^ ^]^ The log P values of ligands were analyzed with the SYBYL‐X software (Tripos Inc., St. Louis, MO, USA). The complex structures were drawn with CYLview (www.cylview.org). The unit of binding energy is presented in kcal mol^−1^.

### Cell Culture and Cytotoxicity Assay

HepG2, NCTC, C2C12, L929, and HEK‐293T cells were obtained from the Shanghai Cell Bank of Type Culture Collection of the Chinese Academy of Sciences (Shanghai, China). Cells were cultured in Dulbecco's modified Eagle's medium (DMEM, Corning, USA) supplemented with 10% fetal bovine serum (FBS, Gibco, USA) and 1% penicillin‐streptomycin (Corning, USA) in a humidified incubator at 37 °C and with 5% CO_2_. For cytotoxicity assay, 1.0 × 10^4^ cells were seeded in 96‐well plates and afterward cultured overnight. Subsequently, cells were treated with different chemicals at various concentrations, followed by cytotoxicity assessments after 24 h. Cell viability was determined through the CCK‐8 assay following the standard protocol from the manufacturers (Solarbio, 1000 T, China). For cell death determination, cells were seeded in 6‐well plates at a density of 1.0 × 10^5^ cell well^−1^ and were then cultured overnight. Then, cells were washed with PBS for three times and afterward treated with different compounds at the concentration of 20 µm for 24 h. After washing with PBS for three times, cells were stained with PI at 10 µm in the ice for 15 min. Eventually, cells were analyzed on an LSR II Flow Cytometer (BD, USA).

### In Vitro Prussian Blue Stain

Cells were seeded in 35 mm petri dishes at a density of 3–4 × 10^5^ cells well^−1^ and were then cultured overnight. Cells were supplemented with 100 µm FeCl_3_ for 3 h, followed by treatment with DFO, DFX, and **DFA1** for 12 h. After washing with PBS three times and then fixing with 4% paraformaldehyde for 30 min, cells were washed three times with PBS and then stained with Prussian blue for 30 min. After staining, cells were washed again with PBS and counter‐stained with nuclear fast red. The stained cells were visualized with light microscope (Olympus BX53).

### Fluorescent Microscopy Characterization

HepG2 and NCTC cells were first seeded in 35 mm petri dishes at a density of 3–4 × 10^5^ cells well^−1^ and were then cultured overnight. Cells were pretreated with 100 µm FeCl_3_ for 3 h, followed by treatment with different compounds for additional 12 h. After washing with PBS three times, cells were stained with Hoechst 33342 (10 µm) and FerroOrange (1 µm) (Dojindo Molecular Technologies, Inc, Japan) in PBS at 37 °C for 20 min. After washing with PBS for three times, the fresh culture medium was supplemented for fluorescence microscopy imaging (DMI6000, Leica).

### Flow Cytometry Analysis of Intracellular Labile Iron Pool

First, HepG2 and NCTC cells were seeded in 6‐well plates at a density of 1.0 × 10^5^ cell well^−1^, and were then cultured overnight. Afterward, the cells were pretreated with 100 µm FeCl_3_ for 3 h, followed by washing with PBS for three times, and further treated with different compounds at 20 µm for 12 h. After washing with PBS for three times, cells were stained with Ca‐AM at 10 µm at 37 °C for 30 min. Finally, after washing with PBS for three times, cells were re‐suspended in PBS for flow cytometry analysis on an Novocyte Flow Cytometer (USA).

To assess lipid peroxidation, HepG2 and NCTC cells were exposed to 100 µm FeCl_3_ and incubated for 12 h at 30 °C. Pretreated cells were washed with PBS for three times and then treated with DFO, DFX, and **DFA1** at the concentration of 20 µm for 12 h. After washing with PBS for three times, cells were stained with 10 µm C11‐BODIPY^581/591^ (Invitrogen, USA) for 2 h. The treated cells were washed three times by PBS and re‐suspended for flow cytometry analysis on an Novocyte Flow Cytometer (USA).

### Immunofluorescent Staining

After treatment, cells were fixed in 4% paraformaldehyde, and were then permeabilized with PBST containing 0.25% Triton‐X‐100. Thereafter, cells were immersed in 2% BSA in PBST to block the nonspecific background, and were then subjected to incubation with anti‐4‐HNE Ab (Abcam, 1:100) at 4 °C overnight. After washing with PBS three times, the secondary Ab conjugated with TRITC/Rhodamine was applied to cells, followed by incubation for 1 h at room temperature. Eventually, cells were re‐washed with PBS, coverslip‐mounted (with DAPI), and examined through fluorescence microscopy imaging (DMI6000, Leica). For tissue immunofluorescence, liver sections were deparaffinized and rehydrated. Then, cells were washed with PBS and immersed in 3% BSA to block the nonspecific background. The sections were then incubated with rabbit polyclonal anti‐4‐HNE primary Ab (Abcam, 1:100) at 4 °C overnight. After washing for three times with PBS, secondary Ab conjugated with TRITC/Rhodamin was applied to the sections, followed by incubation for 1 h at room temperature. The sections were rewashed with PBS, coverslip‐mounted (with DAPI), and observed by fluorescence microscopy imaging (DMI6000, Leica).

### Animal Experimentation

All animal experiments were approved by the Animal Ethics Committee at the Research Center for Eco‐Environmental Sciences, Chinese Academy of Sciences. Balb/c mice (6 weeks old) and C57BL/6 (4 weeks old) mice were purchased from the Vital River Laboratory Animal Technology Co. Ltd (Beijing, China). *Hfe^–/–^* mice on the 129S background were generously provided by Dr. Fudi Wang.^[^ ^46^ ^]^ All mice were bred in a specific pathogen‐free (SPF) facility. Compounds were dissolved in DMSO to prepare the stock solution (5.7% DMSO, 9.6% Cremopho EL and 9.6% ethanol in PBS) and were injected through i.v. route at a dose of 30 mg kg^−1^ body weight. To establish iron overload mice, wild‐type mice were intraperitoneal (i.p.) injection of iron‐dextran (150 mg kg^−1^) every other day for 1 week, followed by treatment with compounds (5.7% DMSO, 9.6% Cremopho EL and 9.6% ethanol in PBS) through i.v. injection at a dose of 30 mg kg^−1^ body weight. For the oral administration, compounds were dissolved in DMSO to prepare the stock solution, and were then diluted with 70% propylene and 30% saline. Thereafter, administration of compounds was implemented through the oral route at a dose of 20 mg kg^−1^ body weight. For dietary high‐iron treatment, C57BL/6 mice (4 weeks old) were fed on a high‐iron diet containing 8.3 g carbonyl iron per kg body weight (Research Diets, Inc) for 8 weeks. After treatment, mice were sacrificed at different time points, and various tissues were harvested for further experiments.

### Plasma Half‐Life of DFO and DFA1

Balb/c mice (6 weeks old) were i.v. injected via the tail vein with DFO and **DFA1** at a dose of 100 mg kg^−1^ body weight. At various time intervals (5, 20, 30 min, 1, 2, 6, 12, and 24 h), mice were euthanized for specimen collection. Plasma was separated by centrifuging samples at 2000 rpm for 15 min. Acetonitrile (100%, 0.3 mL) was added to precipitate the protein in the plasma. The mixture was centrifuged at 10, 000 g, and the concentrations of DFO and **DFA1** in the supernatant were determined by HPLC (SPD‐20A/20AV Series with a SIL‐20A/20AC detector). The samples were injected into a reverse‐phase C‐18 column and eluted with a mobile phase consisting of methanol‐water (30:70 v/v) at a flow rate of 1.0 mL min^−1^. The plasma concentrations were detected at a wavelength of 430 nm for DFO and 550 nm for **DFA1**. Pharmacokinetic analysis was performed using the two‐compartment model to estimate the pharmacokinetic parameters, including area under the curve, apparent volume and clearance rate, apparent volume of distribution, and peak concentration of the drug.

### Iron Parameter Analyses

Serum iron concentration was measured with a kit following the protocol from the manufacturer (Nanjing Jiancheng Bioengineering Institute, China). Liver and splenic iron content was assessed, as previously described.^[^ ^47^ ^]^ In brief, dissected liver and spleen specimens were subjected to digestion with the mixed acid solution (49.6 hydrochloric acid, 20% saturated trichloroacetic acid, and 30.4% ddH_2_O) at 65 °C for 24 h, followed by ultra‐sonication with cubic zirconia beads and continuous incubation for 48 h. Iron concentration was measured by Chromagen solution. The absorbance was measured with a Varioskan Flash multimode reader at 535 nm (Thermo Fisher Scientific, USA).

### Determination of IL‐6, *β*2M, and Other Markers

Serum IL‐6 concentration was determined by an ELISA assay kit purchased from the USCN (Wuhan, China). *β*2M was detected by ELISA assay kits purchased from Cloud‐Clone Corp (Wuhan, China). Levels of LDH, Cr, AST, and ALT in sera were carried out with kits from the manufacturers (Nanjing Jiancheng Bioengineering Institute, China). MDA was measured by kits from the manufacturers (Solarbio, China).

### Histological Examination and Iron Staining

Following the standard protocols, as described,^[^ ^37a^ ^]^ tissue specimens were first fixed in 4% PBS‐buffered paraformaldehyde solution, and thereafter were embedded in paraffin for sectioning and hematoxylin‐eosin (H&E) staining. For iron staining, deparaffinized tissue sections were treated with 1% hydrogen peroxide (H_2_O_2_) for 30 min to eliminate the activity of endogenous peroxidase. Tissue sections were stained with Prussian blue stain (Solarbio, China) following a standard protocol.^[^ ^48^ ^]^

### Western Blot Analysis

Total proteins were extracted from cells and liver tissues with ice‐cold RIPA lysis buffer (Applygen, China) containing 15% proteinase inhibitor cocktail (Roche). Concentrations of total proteins were assayed using the BCA method (Solarbio, China). Equal mass of total proteins for each sample were subjected to SDS‐PAGE, followed by transfer onto nitrocellulose membranes. Western blotting was carried out following the standard protocols, as previously described.^[^ ^48^ ^]^ Primary Ab was as follows, anti‐*β*‐actin Ab (1:2000 dilution, Proteintech, China), anti‐ferritin light chain Ab (1:1000, dilution, Proteintech, China), anti‐GPX4 Ab (1:1000, dilution, Proteintech, China), anti‐NOX1 Ab (1:1000, dilution, Proteintech, China) and anti‐4‐HNE Ab (1:1000, dilution, ab46545, Abcam).

### Statistical Analysis

All experimental data here are shown as mean ± standard deviation (SD) with sample sizes (n) stated for each case individually. Independent *t*‐test statistical analysis was performed to evaluate the significance of the experimental data. One‐way ANOVA analysis was used to assess the statistical differences among more than two groups. Statistical significance was determined as *P* < 0.05 and *P* < 0.001. All statistical analyses were performed using the SPSS software, version 17.0 (IBM Corp, Armonk NY).

## Conflict of Interest

The authors declare no conflict of interest.

## Author Contributions

W.F., J.Z., and S.L. designed the experiments and drafted the manuscript. W.F., Y.X., C.Z., and Z.Z. performed the experiments and analyzed the results. W.F., C.Z., Z.Z., W.L., T.G., J.Z., and S.L. wrote and revised the manuscript. J.M. and S.L. obtained funding and supervised the study. All authors approved the final version of the paper.

## Supporting information

Supporting Information

## Data Availability

The data that support the findings of this study are available from the corresponding author upon reasonable request.
