# Supplementary material for: New Deferric Amine Compounds Efficiently Chelate Excess Iron to Treat Iron Overload Disorders and to Prevent Ferroptosis
Source: Adv Sci (Weinh). 2022 Aug 28;9(29):2202679. doi: 10.1002/advs.202202679 (PMC9561787; doi:10.1002/advs.202202679)
Supplement: Supplementary file 1 — Supporting Information [file ADVS-9-2202679-s001.pdf]

ADVANCED SCIENCE
Open Access

Supporting Information

for *Adv. Sci.*, DOI 10.1002/advs.202202679

New Deferric Amine Compounds Efficiently Chelate Excess Iron to Treat Iron Overload Disorders and to Prevent Ferroptosis

Wenya Feng, Yuanjing Xiao, Chuanfang Zhao, Zhanming Zhang, Wei Liu, Juan Ma, Tomas Ganz, Junliang Zhang and Sijin Liu**

Supplemental information

New Deferric Amine Compounds Efficiently Chelate Excess Iron to Treat Iron Overload Disorders and to Prevent Ferroptosis

Wenya Feng[1,2], Yuanjing Xiao[3], Chuanfang Zhao[1,2], Zhanming Zhang[4], Wei Liu[1,2], Juan Ma[1,2], Tomas Ganz[5], Junliang Zhang[4,*], Sijin Liu[1,2,*]

1. State Key Laboratory of Environmental Chemistry and Ecotoxicology, Research Center for Eco-Environmental Sciences, Chinese Academy of Sciences, Beijing 100085, China.
2. University of Chinese Academy of Sciences, Beijing 100049, China
3. School of Chemistry and Molecular Engineering, East China Normal University, 500 Dongchuan Road, Shanghai 200241, China.
4. Department of Chemistry, Fudan University, 2005 Songhu Road, Shanghai 200438, China.
5. Department of Medicine, David Geffen School of Medicine, University of California, Los Angeles, CA 90095, USA.

*: correspondence to Sijin Liu, Ph.D, email: sjliu@rcees.ac.cn

Junliang Zhang, Ph.D, email: junliangzhang@fudan.edu.cn

Supplemental Methods

Procedures for the synthesis of deferric amine compounds

Step 1: Synthesis of (*R*, *E*)-N-(3,5-di-tert-butyl-2-hydroxybenzylidene)-2-methylpropane-2-sulfinamide (*Rs*)-**3**. To obtain the *o*-hydroxyl benzyl amine compounds, a series of intermediates were synthesized as follows. Briefly, a solution of the sulfinamide (2.66 g, 22 mM) and 3,5-di-tert-butyl-2-hydroxybenzaldehyde (4.69 g, 20 mM) in THF (60 mL) were added with $Ti(OPr^{i})_4$ (11.37 g, 2.0 equiv), and the reaction mixture was heated to 50 °C and continuously stirred for 18 h. Upon reaction completion, the reaction mixture was allowed to cool down to room temperature, and were then diluted with ethyl acetate (EtOAc), and poured into brine with rapid stirring. The resulting suspension was filtered through a plug of Celite, and the filter cake was washed with EtOAc. The filtrate was afterwards transferred to a separatory funnel, and the organic layer was washed with an equal volume of brine. The organic phase was dried with $MgSO_4$, filtered, concentrated, and purified by flash chromatography (PE: EA = 10:1) to afford chiral imine (*Rs*)-**3** (4.75 g, 70%), as described[1].

Step 2: Synthesis of (*R*)-N-((*S*)-(3,5-di-tert-butyl-2-hydroxyphenyl)-2-methylpropane-2-sulfinamide (*S*, *Rs*)-**4**. In the following, a solution of (*Rs*)-3 (10 mM) in THF (20 mL) at - 48 °C was added dropwise with Grignard reagent (3.0 equiv, 30 mM). The mixture was stirred at - 48 °C for 4-6 h and then was warmed to room temperature with stirring overnight. When completed according to the TLC analysis, the reaction mixture was quenched by the addition of NH_4Cl aqueous (aq). and diluted

with EtOAc. The organic layer was separated, and the aqueous layer was extracted twice with EtOAc (40 mL×3). The combined organic layers were dried over Na_2SO_4, filtered, concentrated, and purified by flash chromatography to obtain (*S*, *Rs*)-**4**.

Step 3: Synthesis of (*S*)-2-(aminomethyl)-4,6-di-tert-butylphenol. Thereafter, 3 M HCl (5 mL) was added into the solution of the above (*S*, *Rs*)-**4** (5 mM) in MeOH, and the reaction mixture was stirred at room temperature for 3 h until the completion of material, as characterized by the TLC analysis. Solvent MeOH was removed in vacuo, and 20 mL EtOAc was in turn added into the above residue. Then, the saturated potassium bicarbonate was added slowly to the solution until bubbles were no longer formed. The organic layers were separated and extracted 3 times with 20 mL EtOAc. The combined organic phases were washed with brine and dried over Na_2SO_4, filtered, concentrated, to afford yellow crude solid without purification for next the step.

Step 4: Synthesis of **DFA1-DFA4**. Anhydrous Na_2SO_4 (2.5 equiv, 15 mM) and the aldehyde (6 mM) were added into the solution of the above residue in MeOH (25 mL), and the reaction mixture was heated to 70 °C and stirred for 12 h until the completion of material (monitored by TLC). The reaction mixture was cooled to 0 °C, and $NaBH_3CN$ (15 mM) was added portion-wise. The reaction mixture was stirred for 3 h until intermediate was consumed (monitored by TLC), followed by filtering and concentration. And the residue was directly purified by chromatography to obtain aminophenol ligands **DFA1-DFA4**.

(*S*)-2,4-di-tert-butyl-6-(1-((2-hydroxy-5-methylbenzyl)amino)ethyl)phenol (**DFA1**). Yield = 72%. Yellow solid. Mp 89.6 – 91.5 °C. $[\alpha]_D^{20}$ = 12.6 (c = 0.25, $CHCl_3$). ^1H NMR (500 MHz, $CDCl_3$) δ 7.24 (d, *J* = 1.6 Hz, 1H), 6.98 (d, *J* = 8.0 Hz, 1H), 6.89 (s, 1H), 6.83 (d, *J* = 1.1 Hz, 1H), 6.72 (d, *J* = 8.1 Hz, 1H), 3.94 (q, *J* = 6.5 Hz, 1H), 3.73 (dd, *J* = 106.6, 13.0 Hz, 2H), 2.26 (s, 3H), 1.47 (s, *J* = 5.4 Hz, 12H), 1.32 (s, 9H). ^{13}C NMR (125 MHz, $CDCl_3$) δ 153.45 (s), 152.18 (s), 140.65 (s), 135.94 (s), 131.49 (s), 129.82 (s), 129.37 (s), 125.40 (s), 124.25 (s), 123.12 (s), 122.56 (s), 115.82 (s), 58.78 (s), 47.24 (s), 34.96 (s), 34.16 (s), 31.70 (s), 29.72 (s), 22.37 (s), 20.37 (s). HRMS(ESI) calcd for $C_{24}H_{36}NO_2$: 370.2741, found: 370.2741.

(*S*)-2-(1-(((1H-indol-2-yl)methyl)amino)ethyl)-4,6-di-tert-butylphenol (**DFA2**). Yield = 65%. White solid. Mp 107.2 – 109.3 °C. $[\alpha]_D^{20}$ = 35.8 (c = 0.25, $CHCl_3$). ^1H NMR (500 MHz, $CDCl_3$) δ 8.06 (s, 1H), 7.70 (m, *J* = 7.8 Hz, 1H), 7.39 (d, *J* = 8.1 Hz, 1H), 7.22 (d, *J* = 11.1 Hz, 2H), 7.16 (t, *J* = 7.4 Hz, 1H), 7.11 (s, 1H), 6.86 (s, 1H), 4.02 (m, 2H), 3.88 (d, *J* = 13.4 Hz, 1H), 1.49 (s, 9H), 1.46 (d, *J* = 6.7 Hz, 3H), 1.33 (s, 39H). ^{13}C NMR (125 MHz, $CDCl_3$) δ 154.02 (s), 140.17 (s), 136.24 (s), 135.88 (s), 123.11 (s), 122.96 (s), 122.46 (s), 122.33 (s), 119.78 (s), 118.84 (s), 113.93 (s), 111.18 (s), 59.44 (s), 42.18 (s), 35.03 (s), 34.16 (s), 31.75 (s), 29.70 (s), 22.46 (s). HRMS(ESI) calcd for $C_{25}H_{35}N_2O$: 379.2744, found: 379.2749.

(*S*)-2,4-di-tert-butyl-6-(1-((cyclohexylmethyl)amino)ethyl)phenol (**DFA3**). Yield = 87%. White solid. Mp 110.2 – 110.9 ^oC. $[\alpha]_D^{20}$ = - 9.8 (c = 0.25, $CHCl_3$). ^1H NMR (400 MHz, $CDCl_3$) δ 11.86 (s, 1H), 7.18 (d, *J* = 2.3 Hz, 1H), 6.80 (d, *J* = 2.2 Hz, 1H),

3.85 (q, J = 6.7 Hz, 1H), 2.59-2.37 (m, 2H), 1.81 (d, J = 12.8 Hz, 1H), 1.76 – 1.62 (m, 4H), 1.45 (d, J = 6.7 Hz, 6H), 1.43 (s, 9H), 1.29 (s, 9H), 1.25-1.12 (m, 3H), 1.02 – 0.87 (m, 2H). ^{13}C NMR (125 MHz, $CDCl_3$) δ 153.88 (s), 139.97 (s), 135.71 (s), 125.89 (s), 122.73 (s), 122.31 (s), 60.13 (s), 54.16 (s), 37.98 (s), 34.94 (s), 34.12 (s), 31.72 (s), 31.31 (s), 31.19 (s), 29.62 (s), 26.51 (s), 26.02 (s), 25.93 (s). HRMS(ESI) calcd for $C_{22}H_{40}NO$: 346.3104, found: 340.3105.

(*S*)-2,4-di-tert-butyl-6-(2,2-dimethyl-1-((naphthalen-1-ylmethyl)amino)propyl)phenol (**DFA4**). Yield = 81%. White solid. ^{1}H NMR (400 MHz, $CDCl_3$) δ 12.03 (s, 1 H), 8.04 (d, J = 8.1 Hz, 1 H), 7.87 (d, J = 7.8 Hz, 1 H), 7.80 (d, J = 8.0 Hz, 1 H), 7.51 (p, J = 7.0 Hz, 2 H), 7.45-7.37 (m, 2 H), 7.23 (d, J = 2.7 Hz, 1 H), 6.78 (d, J = 2.7 Hz, 1 H), 4.30 (dd, J = 12.7, 9.9 Hz, 1 H), 4.03–3.98 (m, 1 H), 3.53 (d, J = 2.4 Hz, 1 H), 2.23 (dd, J = 10.3, 4.6 Hz, 1 H), 1.48 (s, 9 H), 1.32 (s, 9 H), 0.89 (s, 9 H). ^{13}C NMR (101 MHz, $CDCl_3$) δ 154.77, 138.83, 135.69, 134.69, 133.94, 131.77, 128.93, 128.38, 127.52, 126.81, 126.55, 125.87, 125.54, 123.27, 122.33, 120.35, 75.14, 50.12, 36.16, 34.99, 34.05, 31.77, 29.69, 27.33. HRMS(ESI) calcd for $C_{30}H_{42}NO$: 432.3266, found: 432.3261.

Step 5: Synthesis of (*S*)-N-((3,5-di-tert-butyl-2-hydroxyphenyl)methyl)-amide **DFA5**-**DFA7**. Afterwards, the solution of organic acid (1.2 equiv., 1.2 mM), BoP (1.25 equiv., 1.25 mM) and Et_3N (6.0 equiv., 6 mM) in THF (8 mL) was added with the solution of the above (*S*)-2-(aminomethyl)-4,6-di-tert-butylphenol and in THF (2 mL) at 0 °C. The reaction mixture was stirred at 0 °C for 1 h and then was warmed to room

temperature with 12 h. When completed according to the TLC analysis, the reaction mixture was quenched by the addition of $NaHCO_3$ aq. (5 mL) and diluted with EtOAc. The organic layer was separated, and the aqueous layer was extracted twice with EtOAc (5 mL×3). The combined organic layers were dried over Na_2SO_4, filtered, concentrated, and purified by flash chromatography to obtain the product **DFA5-DFA7**.

(*S*)-N-(1-(3,5-di-tert-butyl-2-hydroxyphenyl)-2,2-dimethylpropyl)-2-hydroxybenzamide (**DFA5**). Yield = 73%. White solid. Mp 56.5 – 58.3 °C. $[\alpha]_D^{20}$ = 6.0 (c = 0.25, $CHCl_3$). ^{1}H NMR (500 MHz, $CDCl_3$) δ 12.44 (s, 1H), 8.59 (s, 1H), 7.42 (d, *J* = 7.8 Hz, 1H), 7.38 (t, *J* = 7.8 Hz, 1H), 7.23 (d, *J* = 2.2 Hz, 1H), 7.10 (s, 1H), 6.97 (d, *J* = 8.3 Hz, 1H), 6.87 (t, *J* = 7.6 Hz, 1H), 5.21 (s, 2H), 1.46 (s, 9H), 1.29 (s, 9H), 1.05 (s, 9H). ^{13}C NMR (125 MHz, $CDCl_3$) δ 169.79 (s), 161.59 (s), 134.02 (s), 125.82 (s), 125.15 (s), 122.82 (s), 122.34 (s), 118.64 (s), 99.97 (s), 34.25 (s), 31.63 (s), 31.52 (s), 30.41 (s), 29.60 (s), 27.43 (s), 27.43 (s), 26.90 (s). HRMS(ESI) calcd for $C_{26}H_{37}NaNO_3$: 434.2666, found: 434.2661.

(*S*)-2-((1-(3,5-di-tert-butyl-2-hydroxyphenyl)ethyl)amino)-2-oxoacetate (**DFA6**). Yield = 61 %. White solid. Mp 52.3 – 54.1 °C. $[\alpha]_D^{20}$ = 26.2 (c = 0.25, $CHCl_3$). ^{1}H NMR (500 MHz, $CDCl_3$) δ 7.20 (d, *J* = 2.0 Hz, 1H), 6.90 (d, *J* = 1.6 Hz, 1H), 5.28 (s, 1H), 4.41 - 4.25 (m, 2H), 4.15 (q, *J* = 6.9 Hz, 1H), 1.53 (t, *J* = 8.3 Hz, 3H), 1.39 (s, 9H), 1.35 (t, *J* = 7.1 Hz, 3H), 1.30 (s, 9H). ^{13}C NMR (125 MHz, $CDCl_3$) δ 167.71 (s), 148.88 (s), 142.27 (s), 137.24 (s), 125.37 (s), 122.36 (s), 121.30 (s), 79.39 (s), 61.67 (s), 47.14 (s),

34.96 (s), 34.28 (s), 31.51 (s), 29.70 (s), 23.54 (s), 14.07 (s). HRMS(ESI) calcd for $C_{20}H_{32}NO_3$: 334.2377, found: 334.2372.

(*S*)-N-(1-(3,5-di-tert-butyl-2-hydroxyphenyl)ethyl)-2-(1H-indol-3-yl)acet-amide (**DFA7**). Yield = 79 %. White solid. Mp 167.6 – 169.2 ℃. $[\alpha]_D^{20}$ = 72.9 (c = 0.25, $CHCl_3$). ^1H NMR (400 MHz, $CDCl_3$) δ 9.30 (s, 1H), 8.23 (s, 1H), 7.46 (d, *J* = 7.9 Hz, 1H), 7.41 (d, *J* = 8.2 Hz, 1H), 7.27 (d, *J* = 2.4 Hz, 1H), 7.25 – 7.22 (m, 1H), 7.12 (m, *J* = 12.8, 4.7 Hz, 2H), 6.92 (d, *J* = 2.3 Hz, 1H), 6.04 (d, *J* = 8.0 Hz, 1H), 5.32 (dd, *J* = 14.8, 7.3 Hz, 1H), 3.72 (q, *J* = 17.7 Hz, 2H), 1.45 (s, 9H), 1.37 (d, *J* = 7.0 Hz, 3H), 1.25 (s,9H). ^{13}C NMR (125 MHz, $CDCl_3$) δ 172.91 (s), 151.76 (s), 141.39 (s), 137.95 (s), 136.34 (s), 128.30 (s), 126.72 (s), 124.04 (s), 123.58 (s), 122.71 (s), 120.21 (s), 119.79 (s), 118.67 (s), 111.42 (s), 108.02 (s), 43.09 (s), 35.24 (s), 34.38 (s), 32.89 (s), 31.61 (s), 29.81 (s), 19.60 (s). HRMS(ESI) calcd for $C_{26}H_{34}NNaO_2$: 429.2512, found: 429.2510.

Supplemental figures

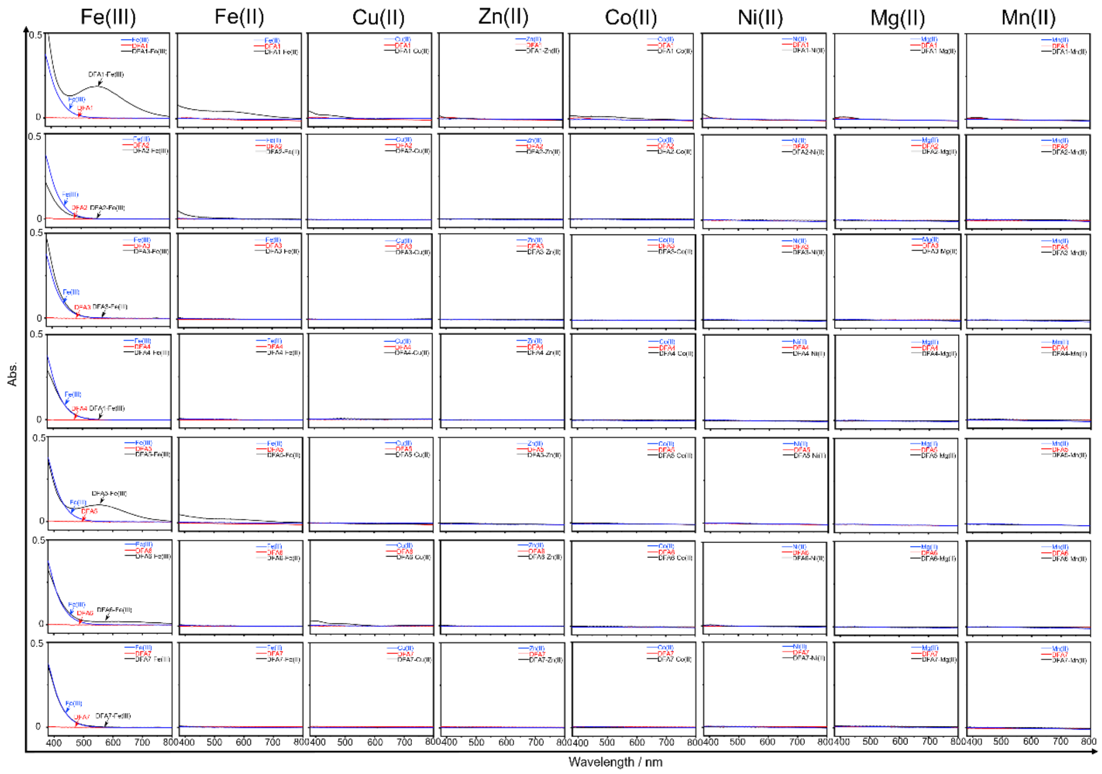

Supplemental Figure 1. Metal selectivity screening of DFAs. The UV-vis absorption spectra of **DFAs** upon incubation with Fe(III), Fe(II), Cu(II), Zn(II), Co(II), Ni(II), Mg(II) and Mn(II).

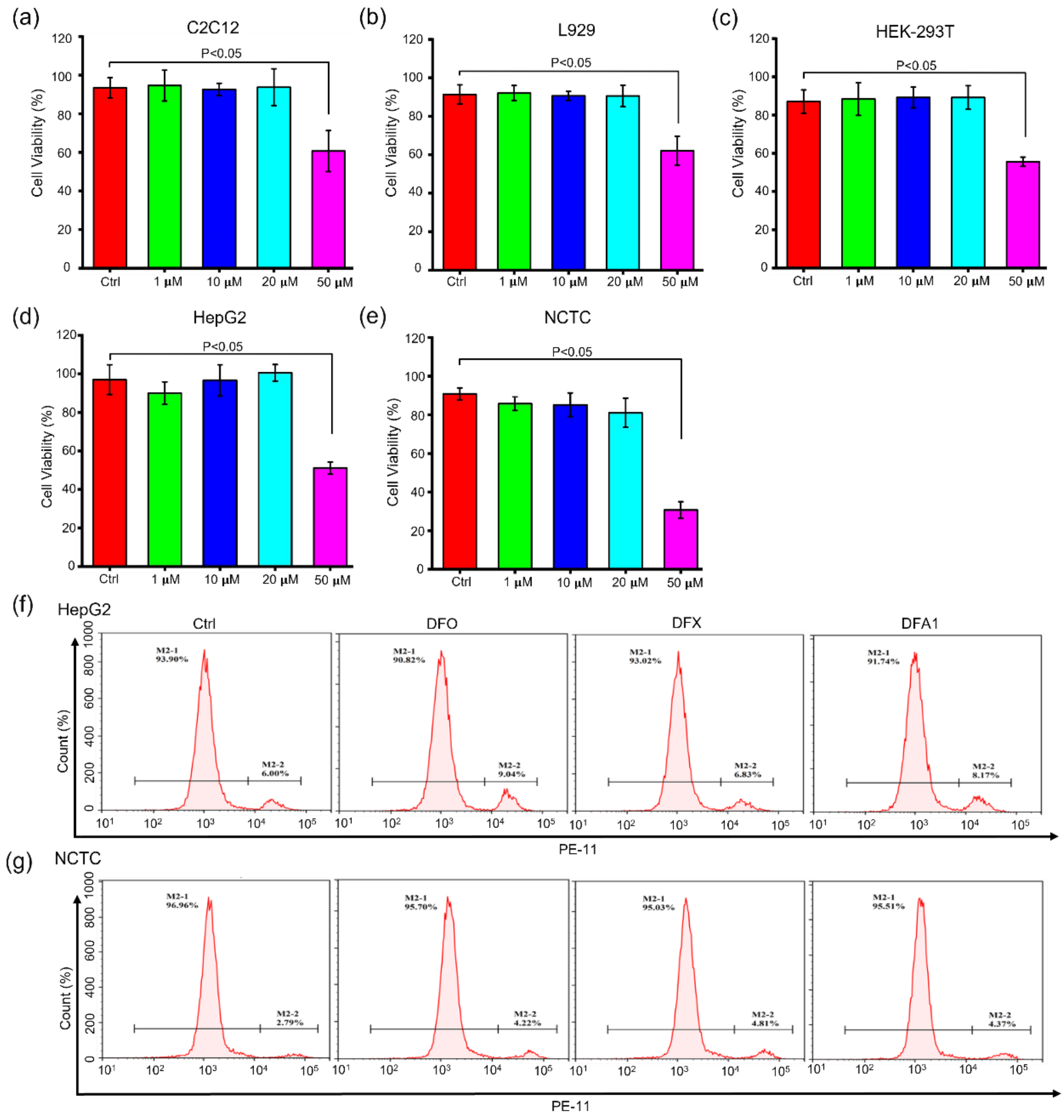

Supplemental Figure 2. *In vitro* cytotoxicity assessment. (a) Cell viability of C2C2, (b) L929, (c) HEK-293T, (d) HepG2 and (e) NCTC cells was assessed through the CCK-8 method after treatment with **DFA1** at different concentrations for 24 h (n=6). Cell death was analyzed through flow cytometry using PI staining in HepG2 (f) and (g) NCTC cells upon treatment at 20 µM for 24 h.

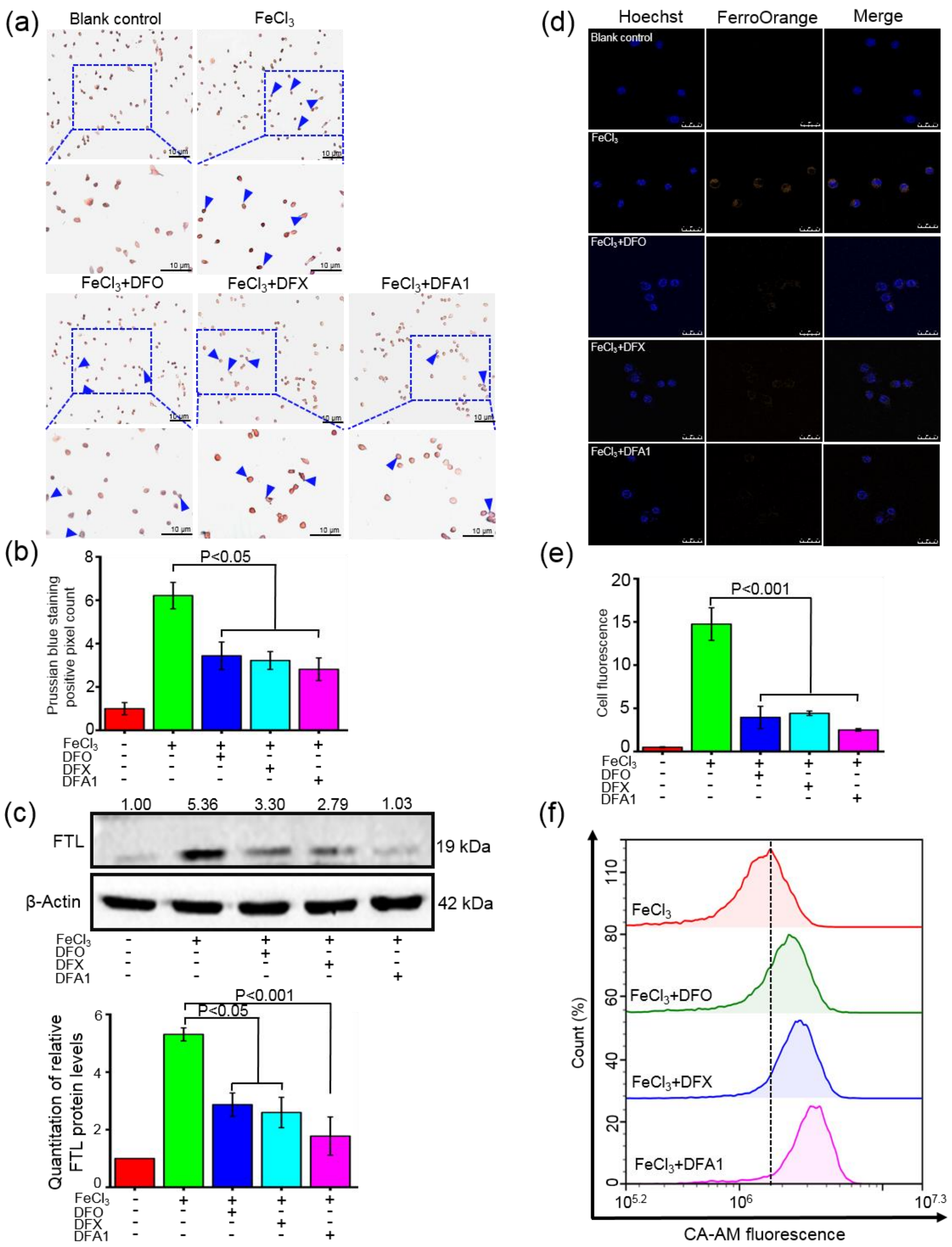

Supplemental Figure 3. Validation of iron chelating efficacy in NCTC cells with $FeCl_3$ pretreatment. (a) Prussian blue staining images of NCTC cells with 3-h per-incubation with $FeCl_3$, followed by treatment with DFO, DFX and **DFA1** for 12 h. The lower panel shows the enlarged images. The quantification of positive pixel counts of

Prussian blue staining in NCTC cells is shown in (b) (n=3). (c) FTL protein levels were determined by Western blotting in the above-treated cells. The ratios of FTL to β-actin were calculated, and the ratio of in the blank control is defined as 1.00. The according ratios are presented above the autoradiograms. Quantified data for multiple biological replicates are shown in the lower panel (n=3). (d) Representative confocal microscopy images showing intracellular ferrous iron in the above-treated cells, as reflected by FerroOrange probes. Cells were stained with FerroOrange probes (in brown color) to visualize the intracellular ferrous iron. Hoechst 33342 was used to stain nuclei (in blue color). Scale bar, 25 μm. Quantified data of cellular fluorescence are shown in (e) (n=3). (f) Assessment of the intracellular ferrous iron mass, namely LIP, using the Ca-AM probes in the above-treated NCTC cells through flow cytometry.

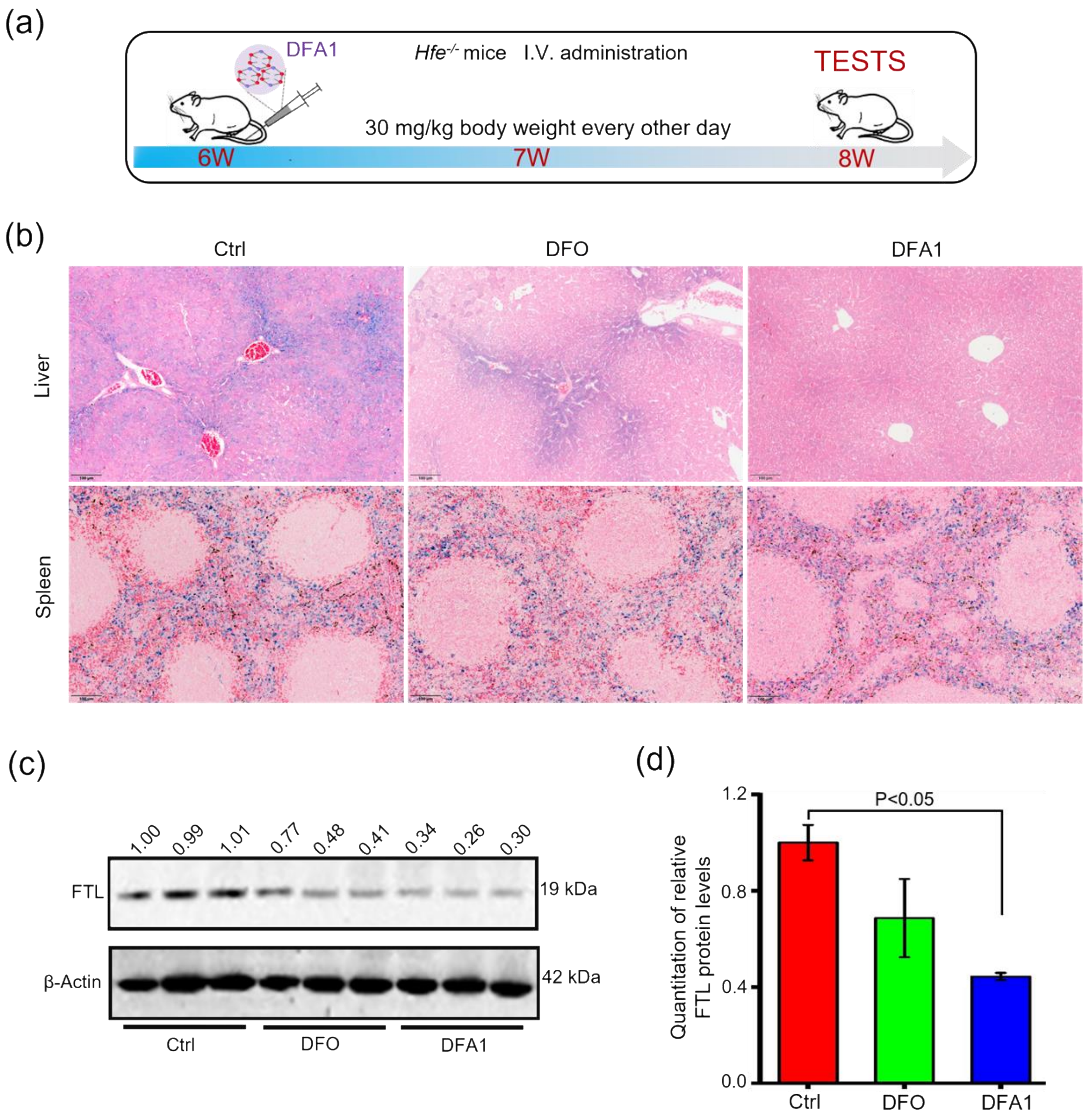

Supplemental Figure 4. Parenteral DFA1 alleviated iron overload in *Hfe*$^{-/-}$ mice. (a) A diagram showing the experimental design. After treatment of *Hfe*$^{-/-}$ mice with DFO and **DFA1** at a dose of 30 mg/kg body weight every other day for 2 weeks, (b) Tissue iron staining of liver and spleen sections with Prussian blue (in blue). Scale bar, 100 μm. (c) FTL protein levels were assayed by Western blot analysis in liver specimens, and quantified data of FTL proteins to the internal control are shown in (d) (n=3).

(a)

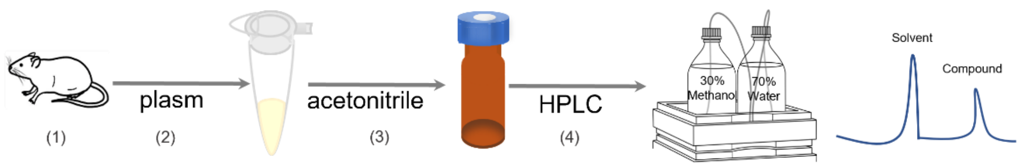

(b)

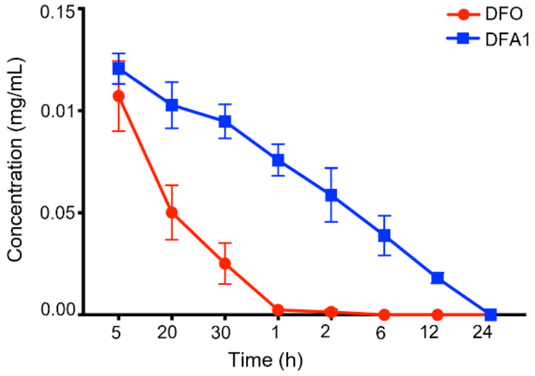

(c)

	DFO (mean ± SD)	DFA1 (mean ± SD)
$t_{1/2\alpha}$ (h)	0.14 ± 0.74	2.01 ± 0.08
$AUC^{0\text{-}t}$ (mg h/L)	6.29 ± 1.85	299.00 ± 38.24
CLz (L/h/kg)	0.02 ± 0.01	0.12 ± 0.01
Vd (L/kg)	0.16 ± 0.25	2.17 ± 0.14
C_{max} (mg/L)	0.12 ± 0.02	0.12 ± 0.01

$t_{1/2\alpha}$: plasm half-life
AUC: area under the curve
CLz: apparent volume and clearance rate
Vd: apparent volume of distribution
C_{max}: peak concentration of drug

Supplemental Figure 5. Pharmacokinetic parameters of DFA1 in mice. (a) A schematic diagram of the experimental design. (b) Plasma concentrations of compound **DFA1** at various time points after tail vein administration of DFO (red) and **DFA1** (blue) (n=5). (c) Analysis of pharmacokinetic parameters of different compounds in mice (n=5).

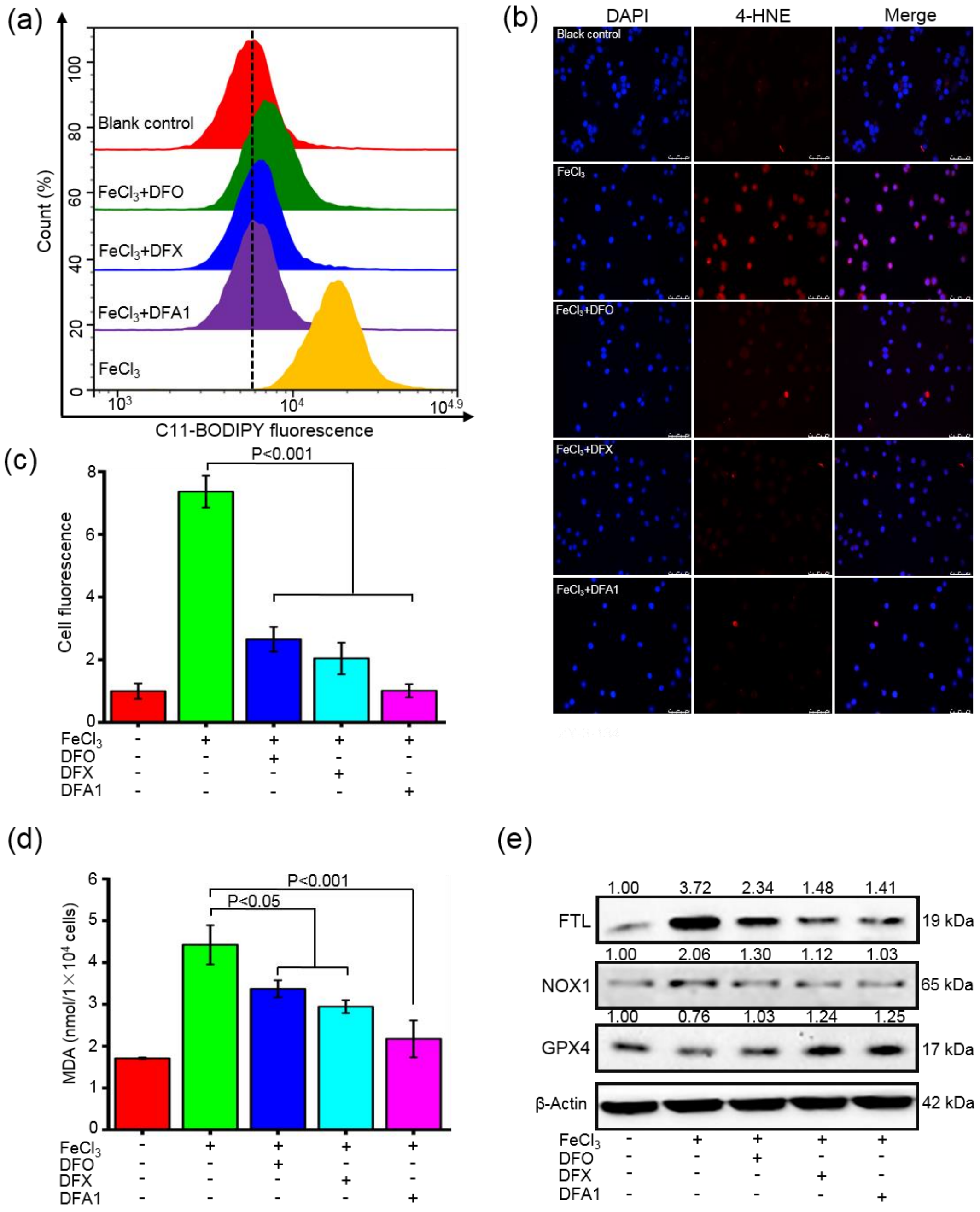

Supplemental Figure 6. DFA1 alleviated ferroptosis in iron-loaded NCTC cells. (a) Determination of lipid peroxidation levels in NCTC cells with pretreatment of $FeCl_3$ at 100 μM for 12 h, followed by treatment with DFO, DFX and **DFA1** at 20 μM for 12 h. Thereafter, lipid peroxidation was assessed with C11-BODIPY$^{581/591}$ probes through flow cytometry. (b) Representative images of 4-HNE immunofluorescent staining (in

red) through fluorescent microscopy. DAPI was used to stain nuclei (in blue). Quantification of cell 4-HNE fluorescence is shown in (c) (n=3). (d) Cellular MDA content was assayed in the above-treated cells (n=4), and (e) protein levels of FTL, NOX1 and GPX4 were examined by Western blotting. The ratios of target proteins to the internal control are shown above the autoradiograms.

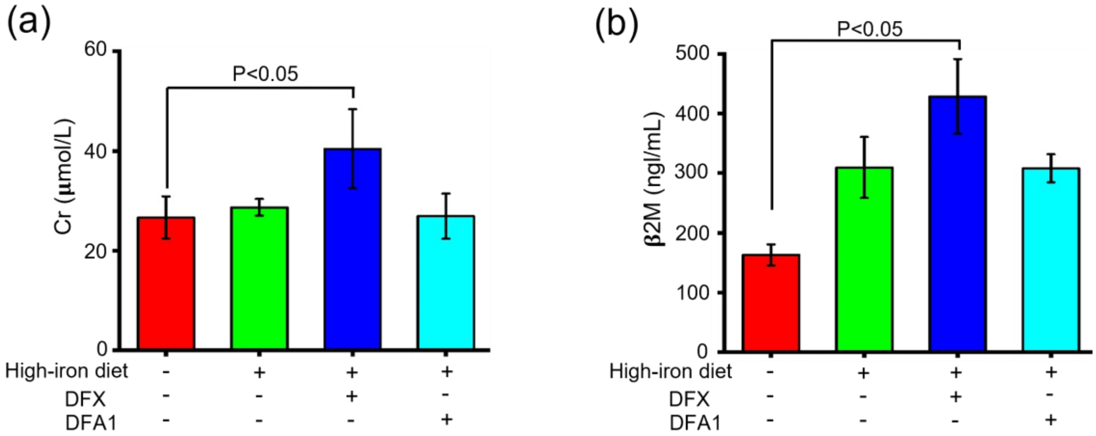

Supplemental Figure 7. Toxicity assessment after oral administration of compounds to mice on high-iron diet. Renal injury biomarker levels of (a) Cr and (b) β2M in high-iron diet C57BL/6 mice responding to oral administration of DFX and **DFA1** at a dose of 20 mg/kg body weight for 4 weeks (n=6-8).

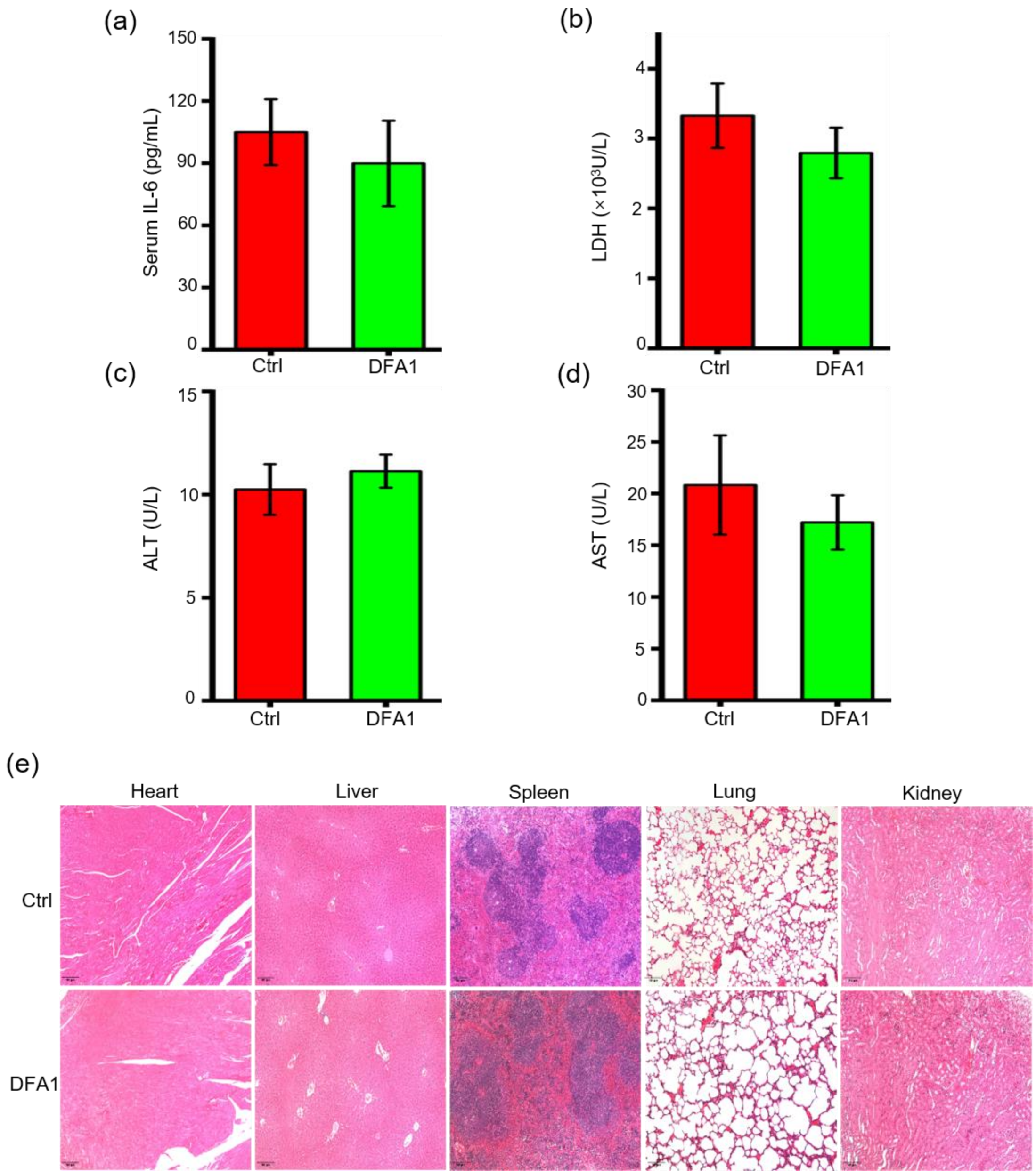

Supplemental Figure 8. Biosafety evaluation of oral administration of DFA1 in *Hfe*$^{-/-}$ mice. (a) Serum IL-6, (b) LDH, (c) ALT and (d) AST levels in *Hfe*$^{-/-}$ mice following oral administration of **DFA1** at a dose of 20 mg/kg body weight for 4 weeks (n=5-6). (e) Histological examination with H&E staining of various organs from *Hfe*$^{-/-}$ mice after treatment. Scale bar, 100 μm.

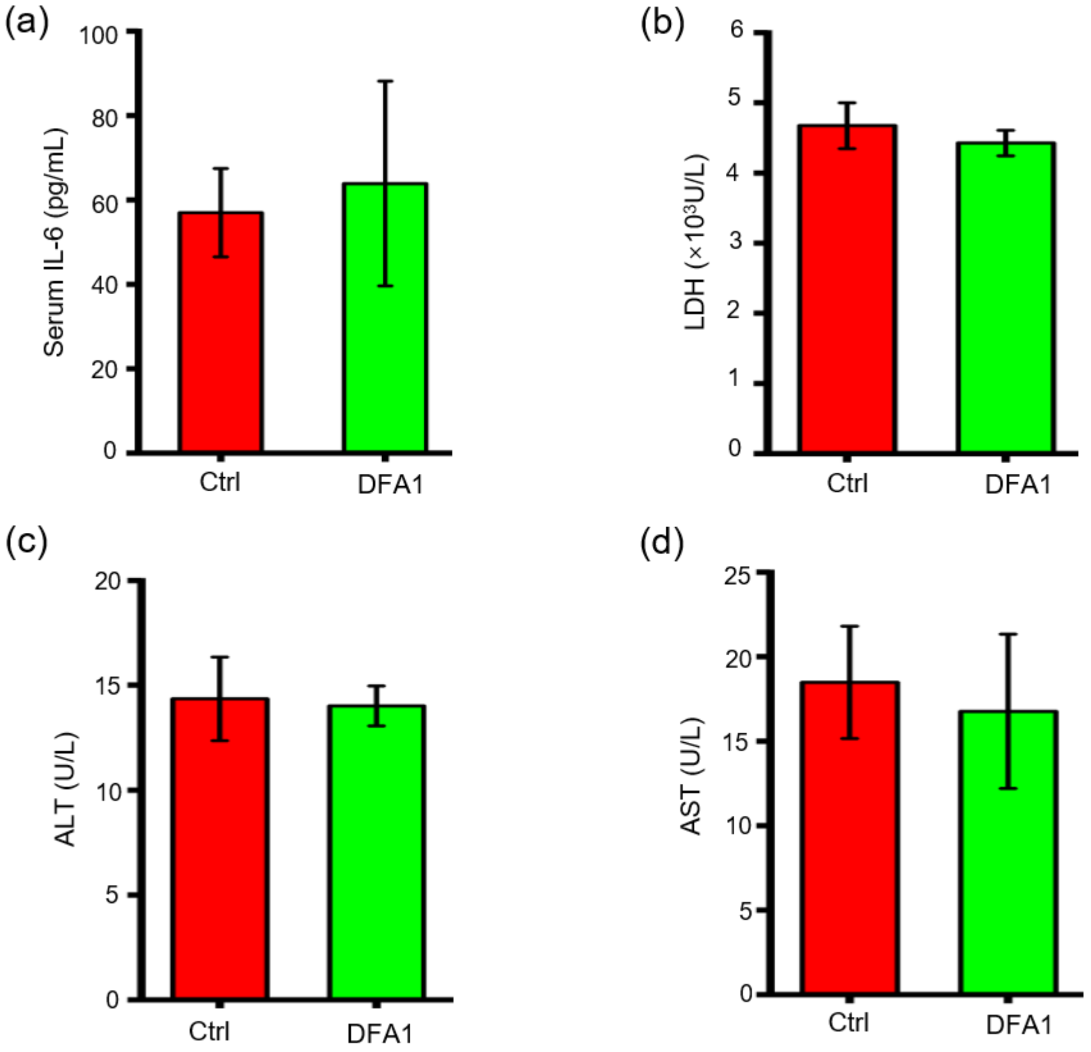

Supplemental Figure 9. Biosafety assessment after tail vein administration of DFA1 to *Hfe*$^{-/-}$ mice. (a) Serum IL-6, (b) LDH, (c) ALT and (d) AST levels in *Hfe*$^{-/-}$ mice following administration of **DFA1** at 30 mg/kg body weight for 2 weeks (n=5-6).

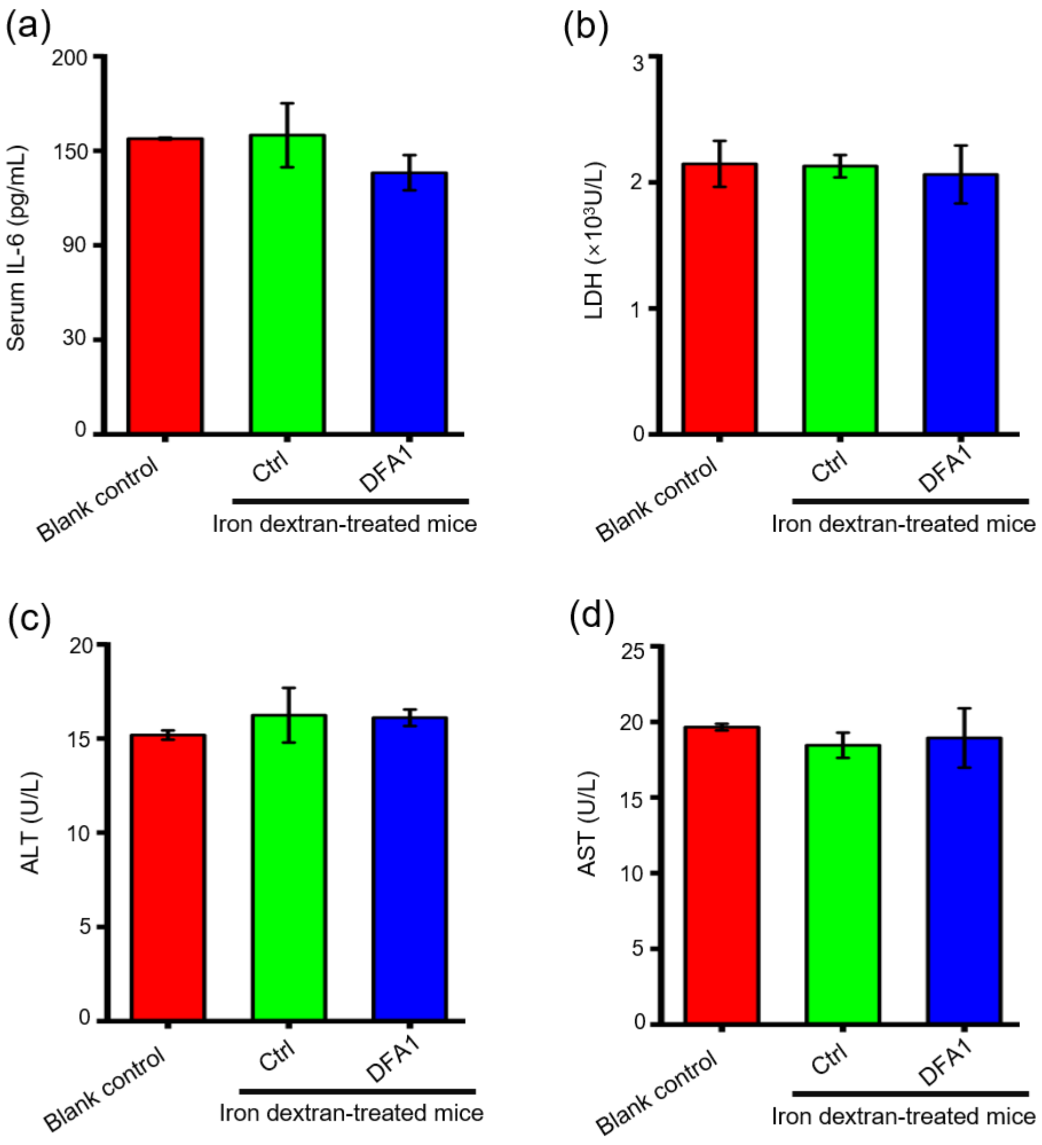

Supplemental Figure 10. Biosafety assessment of tail vein injection of DFA1 to mice with iron overload induced by iron dextran. (a) Serum IL-6, (b) LDH, (c) ALT and (d) AST levels in wild-type mice subjected to iron dextran at 150 mg/kg body weight for a week, followed by administration with **DFA1** at 30 mg/kg body weight every other day for 2 weeks (n=5-6).

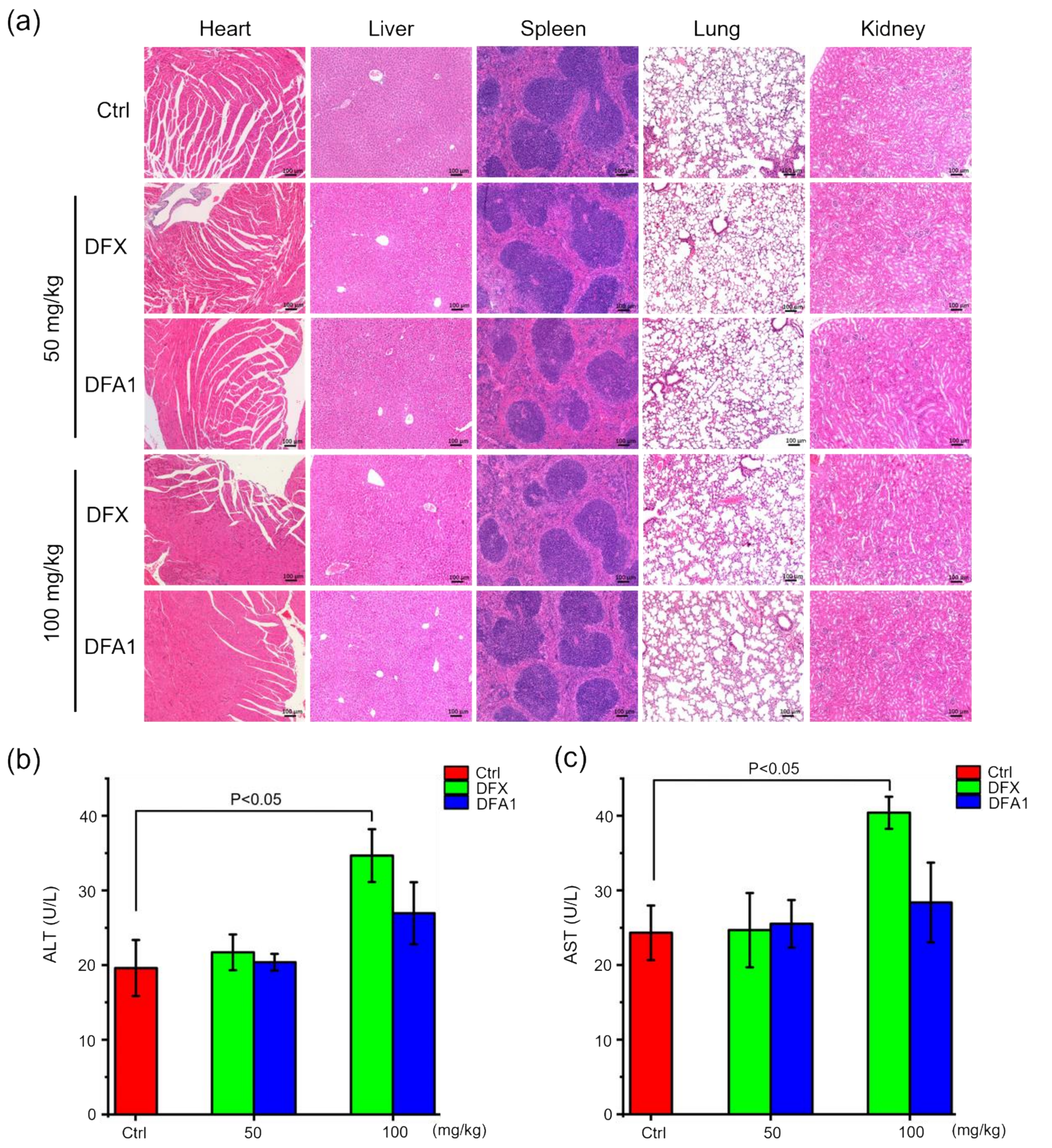

Supplemental Figure 11. Biosafety evaluation of oral administration of DFA1 in wild-type mice. (a) Histological examination with H&E staining of various organs from Balb/c mice following oral administration of DFX and **DFA1** at a dose of 50 or 100 mg/kg body weight for 24 h (n=5-6). Scale bar, 100 μm. (b) ALT and (c) AST levels in these mice after treatment.

Supplemental Table 1. Descriptions of the current iron chelating agents in clinical applications[2-4].

	Deferoxamine (DFO)	Deferiprone (DFP)	Deferasirox (DFX)
Properties	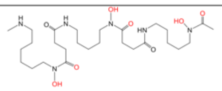	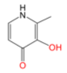	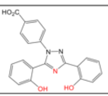
Chelator iron complex	Hexadentate (1:1)	Bidentate (3:1)	Tridentate (2:1)
Daily dose	25-60 mg/kg/day	75-100 mg/kg/day	20-40 mg/kg/day
Administration	Subcutaneous/intravenous, 8-12 h/day	Oral, three times daily	Oral, once daily
Half-life	5-20 min	2-3 h	8-16 h
Indication	Transfusional iron overload	Transfusional iron overload	Transfusional iron overload
Adverse effects	Local reactions at the subcutaneous injection site<br>Growth retardation<br>Bone abnormalities<br>High-frequency sensorineural hearing loss<br>Visual disturbance<br>Neurotoxicity<br>Hypersensitivity reactions	Nausea, abdominal pain, vomiting, diarrhea<br>Neutropenia<br>Agranulocytosis<br>Increased liver enzymes<br>Increased appetite<br>Low plasma zinc level<br>Arthralgia and arthropathy	Nausea, vomiting, abdominal pain, diarrhea and rash<br>Increased serum creatinine level<br>Increased ALT<br>Proteinuria<br>Gastrointestinal disturbances and hemorrhage<br>Genal toxicity<br>Hypoacusis and neurosensory deafness

References

1. Zhang ZM, Chen P, Li W, Niu Y, Zhao XL, Zhang J. A new type of chiral sulfinamide monophosphine ligands: stereodivergent synthesis and application in enantioselective gold(I)-catalyzed cycloaddition reactions. *Angewandte Chemie International Edition*. 2014;53(17):4350-4354.
2. Brittenham GM. Iron-chelating therapy for transfusional iron overload. *New England Journal of Medicine*. 2011;364(2):146-156.
3. Kang H, Han M, Xue J, et al. Renal clearable nanochelators for iron overload therapy. *Nature Communications*. 2019;10(1):5134.
4. Borgna-Pignatti C, Marsella M. Iron chelation in thalassemia major. *Clinical Therapeutics*. 2015;37(12):2866-2877.
